# CircNFIB inhibits tumor growth and metastasis through suppressing MEK1/ERK signaling in intrahepatic cholangiocarcinoma

**DOI:** 10.1186/s12943-021-01482-9

**Published:** 2022-01-17

**Authors:** Jinpeng Du, Tian Lan, Haotian Liao, Xuping Feng, Xing Chen, Wenwei Liao, Guimin Hou, Lin Xu, Qingbo Feng, Kunlin Xie, Mingheng Liao, Xiangzheng Chen, Jiwei Huang, Kefei Yuan, Yong Zeng

**Affiliations:** 1 Department of Liver Surgery & Liver Transplantation, State Key Laboratory of Biotherapy and Cancer Center, West China Hospital, Sichuan University and Collaborative Innovation Center of Biotherapy, Chengdu, 610041 China; 2 Laboratory of Liver Surgery, West China Hospital, Sichuan University, Chengdu, 610041 China

**Keywords:** Intrahepatic cholangiocarcinoma (ICC), Circular RNA, Metastasis, Trametinib, Dual specificity mitogen-activated protein kinase kinase 1 (MEK1)

## Abstract

**Background:**

Considerable evidence shows that circular RNAs (circRNAs) play an important role in tumor development. However, their function in intrahepatic cholangiocarcinoma (ICC) metastasis and the underlying mechanisms are incompletely understood.

**Methods:**

circNFIB (hsa_circ_0086376, termed as cNFIB hereafter) was identified in human ICC tissues through circRNAs sequencing. The biological role of cNFIB was determined in vitro and in vivo by gain or loss of functional experiments. Fluorescence in situ hybridization (FISH), RNA immunoprecipitation (RIP) and RNA pull-down assays were conducted to analyze the interaction of cNFIB with dual specificity mitogen-activated protein kinase kinase1 (MEK1). Duolink in situ proximity ligation assay (PLA) and coimmunoprecipitation (co-IP) assay were used to investigate the effects of cNFIB on the interaction between MEK1 and mitogen-activated protein kinase 2 (ERK2). Finally, a series of in vitro and in vivo experiments were performed to explore the influences of cNFIB on the anti-tumor activity of trametinib (a MEK inhibitor).

**Results:**

cNFIB was significantly down-regulated in human ICC tissues with postoperative metastases. The loss of cNFIB was highly associated with aggressive characteristics and predicted unfavorable prognosis in ICC patients. Functional studies revealed that cNFIB inhibited the proliferation and metastasis of ICC cells in vitro and in vivo. Mechanistically, cNFIB competitively interacted with MEK1, which induced the dissociation between MEK1 and ERK2, thereby resulting in the suppression of ERK signaling and tumor metastasis. Moreover, we found that ICC cells with high levels of cNFIB held the potential to delay the trametinib resistance. Consistently, in vivo and in vitro studies demonstrated that cotreatment with trametinib and lentivirus vector encoding cNFIB showed greater inhibitory effect than isolated trametinib treatment.

**Conclusions:**

Our findings identified that cNFIB played a key role in ICC growth and metastasis by regulating MEK1/ERK signaling. Given the efficacy of cNFIB modulation on ICC suppression and trametinib sensitivity, cNFIB appears to be a potential therapeutic molecule for ICC treatment.

**Supplementary Information:**

The online version contains supplementary material available at 10.1186/s12943-021-01482-9.

## Background

Intrahepatic cholangiocarcinoma (ICC), as the second most common primary hepatic malignancy, accounts for approximately 10–15% of all primary liver cancers [1]. Owing to the increased prevalence of nonalcoholic steatohepatitis and hepatitis C, the incidence of ICC is increasing globally, with an average annual growth of 4.4% over the past 10 years [2]. ICC is generally asymptomatic at the early stage. Most ICC patients are diagnosed at advanced stages, for which limited therapeutic options are available, resulting in poor clinical outcomes. Currently, curative resection remains the cornerstone for cure of ICC, however, 60% of patients who undergo surgery develop recurrent or metastatic disease [3, 4]. Therefore, a better understanding of the molecular mechanism underlying ICC metastasis and identification of new therapeutic targets to suppress metastasis are urgently required for improving the survival outcomes of ICC patients.

Circular RNAs (circRNAs), characterized by single-stranded and covalently closed loop structures, are usually generated by the back-splicing of exons from pre-mRNAs [5]. Previously, circRNAs had been considered as by-products of splicing errors with low abundance. However, through deep RNA sequencing and bioinformatics, circRNAs have been demonstrated as widespread and a substantial presence within transcriptomes [6]. Their prominent features of higher stability than parental linear RNAs, highly conserved expression across species, and tissue- or developmental stage-specific expression suggest that circRNAs may possess multiple biological processes [7, 8]. In addition, emerging studies have proved a vital role of circRNAs in tumor initiation and progression, including hepatocellular carcinoma [9], colorectal cancer [10], glioblastoma [11], and cholangiocarcinoma [12]. Mechanistically, some circRNAs exert function by sponging microRNAs or binding proteins to manipulate gene expression [13, 14], some serve as platforms for protein interaction [15], while some others can encode functional peptides [16]. However, little is known about the contribution of circRNAs in ICC metastasis.

Mitogen-activated protein kinases (MAPK) consist of three major subfamilies: the extracellular-signal regulated kinases (ERK), the c-jun N-terminal kinase or stress-activated protein kinases (JNK or SAPK), and MAPK14 [17]. The ERK signaling pathway, which includes the kinases RAS, RAF, MEK, and ERK, is thought to be a three-tiered or four-tiered phosphorylation cascade that relay upstream signals from membrane receptors to a series of downstream effector substrates [18]. In detail, extracellular signal proteins bind to specific cell-surface receptors, such as cytokine receptors, receptor tyrosine kinases (RTKs) and G protein-coupled receptors, and activate a series of signaling cascades involving RAS, RAF and MEK [19]. As dual-specificity kinases, activated MEK can phosphorylate the conserved threonine and tyrosine residues within the activation loop of ERK, which then regulates some other protein kinases and transcription factors involved in cell proliferation, cell survival, cell migration and cell differentiation [20, 21]. Deregulated activation of, or an enhanced dependence on, RAS/RAF/MEK/ERK pathway is a common feature of many human cancers, including ICC [22–24]. However, whether and how this signaling is regulated by circRNAs remain elusive.

In the current study, through an in-deep analysis of human ICC tissues, we confirmed a circRNA (cNFIB, circBase ID: hsa_circ_0086376) as a tumor suppressor involved in ICC metastasis. Loss of cNFIB favors invasion and metastasis of ICC cells both in vitro and in vivo by activating MEK1/ERK signaling and downstream target genes. Furthermore, cNFIB completely binds to MEK1, thereby impeding ERK phosphorylation and transcriptional activity. More importantly, exogenous overexpression of cNFIB also enhanced anti-tumor effects of trametinib (a specific MEK inhibitor), which implies its promising potential as a therapeutic molecule for combating ICC metastasis.

## Materials and methods

### Human tissues

A total of 222 patients with ICC who underwent curative surgery between October 2010 and December 2017 at West China hospital, Sichuan University (Chengdu, China) were included in this study. The patients were divided into two cohorts. Cohort 1 included 40 patients (20 patients who experienced extrahepatic metastases after surgery and 20 patients who did not experienced metastases after surgery). We chose 30 primary ICC tissues from patients in cohort1 (15 primary ICC tissues from patients with extrahepatic metastases after surgery and 15 primary ICC tissues from patients without postoperative metastases) to perform circRNA-seq. Then the 40 samples (cohort 1) were used for circRNAs validation. Cohort 2 including 182 patients was used for quantification of cNFIB and analysis of the relationship between the expression levels of cNFIB and prognosis of ICC patients. The follow-up period was defined as the interval between surgery and death or recurrence. Overall survival (OS) was defined as the interval from the time of surgery to death. Recurrence-free survival (RFS) was defined as the time from surgery until the detection of any types of recurrence. Patients alive or without recurrence at the time of last follow-up visit were censused. Patients were divided into high and low cNFIB expression groups according to a median cut-off value. All samples and related information from patients in this study were collected with informed consent, and this study was approved by the Institutional Ethics Committee of West China hospital.

### Cell lines and cell culture

All human ICC cell lines (HuCCT1, HCCC9810, and RBE) were purchased from Cell Bank of the Shanghai Institute for Biological Sciences (Chinese Academy of Sciences, Shanghai, China). The three cell lines were maintained in RPMI-1640 medium with 10% fetal bovine serum (HyClone, USA). They were all cultured in a humidified incubator at 37 °C with 5% CO_2_.

### Statistical analysis

The statistical analysis was carried out using SPSS 23.0 and Prism version 7.0 software (GraphPad Software). Data are shown as mean ± standard deviation (S.D). For continuous variables, two-sided Student’s t test was used for two comparisons. One-way ANOVA with Tukey’s post hoc test was used for multiple comparisons. The relationships between cNFIB expression and clinicopathological features of ICC patients were calculated by χ^2^ test or Fisher’s exact test, while the correlation between cNFIB and p-ERK expression was analyzed by Pearson’s correlation test. Survival data were measured by the Kaplan-Meier method and analyzed by the Log-rank test. *P* values less than 0.05 were considered statistically significant (*, *P* < 0.05; **, *P* < 0.01; ***, *P* < 0.001).

## Results

### Decreased cNFIB expression is correlated with ICC metastasis and poor prognosis

**Fig. 1:**
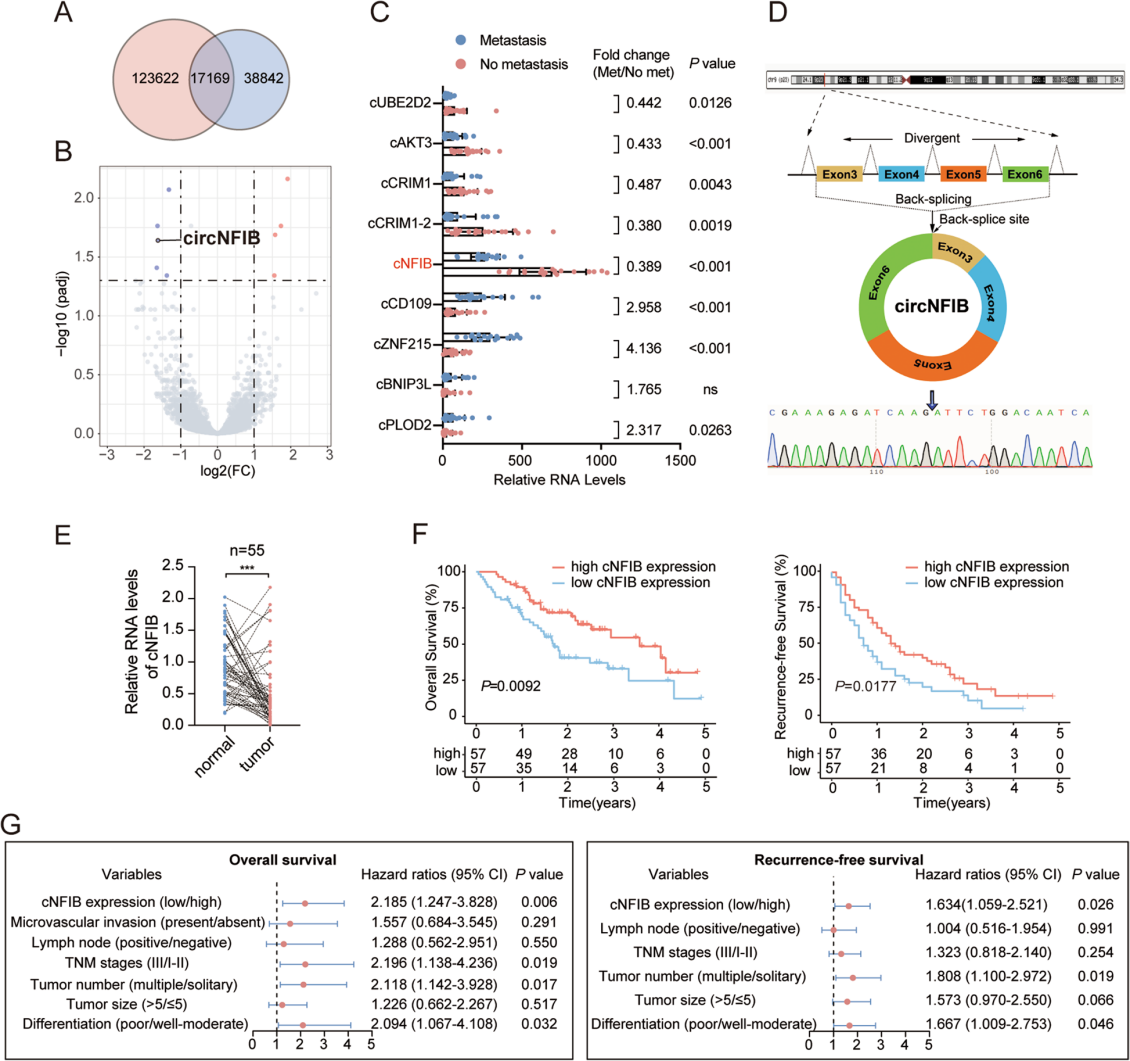
Decreased cNFIB expression is correlated with ICC metastasis and poor prognosis. (**A**) 17,169 circRNAs identified in this study have been documented in circBase. (**B**) Volcano plot of circRNAs that were differentially expressed in 15 ICC tissues with or without extrahepatic metastases identified by circRNA-seq. Vertical dotted lines correspond to up-regulation (Log_2_FC > 1) and down-regulation (Log_2_FC < − 1). Horizontal dotted line corresponds to adjusted *P* = 0.05. (**C**) Results from qRT-PCR validated 3 upregulated and 5 downregulated circRNAs in 20 ICC tissues (cohort1) from patients who experienced postsurgical metastases and 20 ICC tissues from patients that did not (mean ± SD, unpaired Student’s t test). (**D**) Scheme showing the genomic locus and production of cNFIB. Specific divergent primer was designed targeting the back-splice junction and qRT-PCR products were confirmed by Sanger sequencing. (**E**) Relative RNA levels of cNFIB in 55 paired ICC and noncancerous tissues (mean ± SD, paired Student’s t test, ***P < 0.001). (**F**) Kaplan-Meier curves showing the OS (left) and RFS (right) of 114 ICC patients matched by PSM analysis. Patients were stratified by the median expression levels of cNFIB. Log-rank test was used. (**G**) Multivariate analyses showing hazard factors for OS (left) and RFS (right) of the matched cohort. Data were shown as mean ± SD, *P < 0.05; **P < 0.01; ***P < 0.001. Abbreviations: ns, no significance; Met, metastasis; No met, metastasis; TNM, tumor-node-metastasis; CI, confidence interval; ICC, intrahepatic cholangiocarcinoma; OS, overall survival; RFS, recurrence-free survival

In order to screen for essential circRNAs contributing to ICC metastasis, circRNA-seq was performed to analyze the differences of gene expression profiles between 15 primary ICC tissues from patients that experienced extrahepatic metastases after surgery and 15 primary ICC tissues from patients who do not experienced postoperative metastases or recurrence. The clinicopathological features of these 30 patients were shown in Supplementary Table S1. Among the 56,011 circRNAs detected in total, 17,169 (30.65%) circRNAs have been documented in circBase (Fig. 1A). The expression analysis showed that 9 circRNAs were differentially expressed (fold change > 2 (or < 0.5) and adjusted *P* value < 0.05) between the patients that experienced extrahepatic metastases after surgery and those that did not, including 4 upregulated and 5 downregulated circRNAs (Fig. 1B). To further ascertain the RNA-seq results, we designed circRNA-specific divergent primers for the 9 differentially expressed candidates and validated their expression levels by quantitative reverse transcription PCR (qRT-PCR). The Sanger sequencing results demonstrated the PCR products amplified by these primers included the back-splice junctions of the circRNAs (Supplementary Fig. S1A-B). Furthermore, the expression levels of the circRNAs showed no significant changes after treating with ribonuclease R, indicating they were truly circular, not linear (Supplementary Fig. S1C-D). Next, we successfully validated 3 upregulated and 5 downregulated circRNAs in 20 ICC tissues from patients who experienced postsurgical metastases and 20 ICC tissues from patients that did not, which were consistent with circRNA-seq results (Fig. 1C).

cNFIB, derived from exons 3, 4, 5 and 6 of NFIB gene (Fig. 1D), was chosen as a candidate for further study. There were two reasons: (i) The expression levels of cNFIB were the highest among the 9 circRNAs according to its TPM values of RNA-seq data, which were also confirmed by qRT-PCR results from ICC tissues (Fig. 1C). (ii) Previous studies have identified NFIB as a pro-metastatic gene and was highly upregulated in lung cancer and breast cancer [25, 26]. Consistently, the mRNA and pre-mRNA levels of NFIB were much higher in ICC tumor tissues than the paired nontumor tissues (Supplementary Fig. S1E). While, cNFIB was significantly downregulated in primary human ICC tissues compared with normal tissues, as well as in ICC tissues with extrahepatic metastases compared with ICC tissues without metastases (Fig. 1E and C), indicating that lower expression of cNFIB in ICC was suggestive of functionality and was not simply a by-product of splicing. In order to further validate the circular characteristics of cNFIB, HuCCT1 and RBE cell lines were treated with actinomycin D to block the transcription of RNAs. We observed a longer half-life of cNFIB than mNFIB, suggesting the higher stability of cNFIB (Supplementary Fig. S1F). In addition, we extracted RNAs from HuCCT1 and RBE cells, and performed reverse transcription experiments by using oligo (dT)_18_ and random hexamer primers. Significant decrease of cNFIB and no changes of mNFIB expression indicated that cNFIB had no poly-A tail (Supplementary Fig. S1G).

To further explore the relationship between cNFIB expression and clinicopathological features of ICC patients, we expanded our study to 182 tumor tissues. According to the median expression levels of cNFIB, we divided the 182 ICC patients into a low-cNFIB-expression group (*n* = 91) and a high-cNFIB-expression group (n = 91). Notably, a lower cNFIB expression was found to be associated with multiple tumor number, advanced TNM tumor stage, presence of lymph node metastasis and poorer tumor differentiation, indicating that cNFIB involved in ICC progression (Table 1). We next investigated the correlation between of cNFIB levels and survival outcomes of ICC patients. To avoid the non-random assignment of patients, patients were propensity matched 1:1 into low-cNFIB-expression and high-cNFIB-expression group by using Propensity Score Matching (PSM) analysis. All variables presented in Table 1 were used for matching with a caliper equal to 0.05. After matching, there were no significant differences in demographic or tumor-related variables between the two groups (Supplementary Table S2). Consistently, patients with lower cNFIB expression had worse overall survival (OS) and recurrence-free survival (RFS) (Fig. 1F). We next performed univariate and multivariate analysis to identify the risk factors for OS and RFS of the matched cohort. Firstly, we found that tumor number, tumor size, tumor differentiation, lymph node metastasis, TNM stage, and cFNIB expression levels were related to OS or RFS in the univariate analysis (Supplementary Table S3). In the multivariate analysis, decreased cNFIB expression was considered as an independent risk factor for OS, together with poorer tumor differentiation, multiple tumor number and advanced TNM stage. Moreover, we also identified lower cNFIB expression, poorer tumor differentiation, and multiple tumor number as independent risk factors for RFS (Fig. 1G, Supplementary Table S4). Taken together, these results demonstrated that cNFIB was frequently down-regulated in patients that experienced metastasis, pointing to a potential role of cNFIB in ICC progression and metastasis.

**Table 1:** Clinical characteristics of 182 ICC patients (unmatched) based on cNFIB expression levels

Variables	Low cNFIB (*n* = 91)	High cNFIB (n = 91)	*P* value
Age, year, > 60/≤60	46/45	37/54	0.1804
Gender, male/female Ascites, present/absent	54/37 8/83	46/45 11/80	0.2333 0.4671
Hepatolithiasis, present/absent	3/88	2/89	0.6502
HbsAg, positive/negative	28/63	23/68	0.4092
CA19–9, > 22/≤22	65/26	56/35	0.1576
Tumor size (cm), > 5/≤5	54/37	47/44	0.2965
Tumor number, multiple/solitary	38/53	20/71	**0.0042**
Differentiation, poor/well-moderate	73/18	58/33	**0.0133**
MVI, present/absent	12/79	8/83	0.3431
Lymph node, positive/negative	22/69	11/80	**0.0343**
Cirrhosis, with/without	7/84	11/80	0.3206
TNM stage, III/I- II	70/21	49/42	**0.0011**

*ICC* intrahepatic cholangiocarcinoma; *MVI* microvascular invasion; *TNM* tumor-node-metastasis

### cNFIB suppresses ICC growth and metastasis in vitro and in vivo

**Fig. 2:**
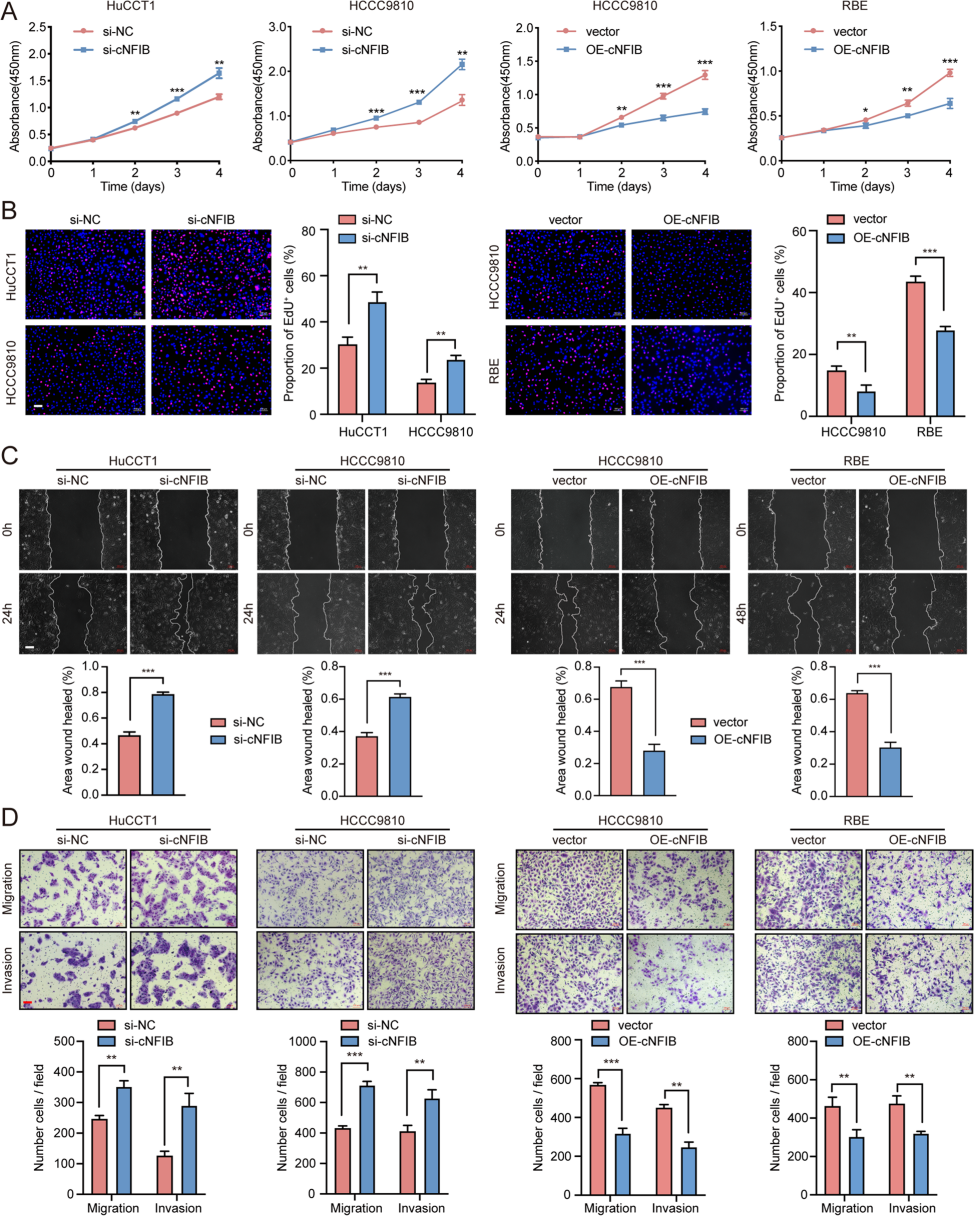
cNFIB suppresses proliferation and metastasis of ICC cells in vitro. (**A**) Cell Counting Kit-8 (CCK-8) assays showed that cNFIB inhibited the proliferation of ICC cells. (**B**) EdU immunofluorescence assays to detect the effects of cNFIB on the capacity of DNA duplication of ICC cells . (**C**) Wound healing showed that cNFIB suppressed the migration of ICC cells. (**D**) Effects of cNFIB on cell migration and invasion examined by transwell assays. Data were shown as mean ± SD, unpaired Student’s t test, *P < 0.05; **P < 0.01; ***P < 0.001. Scale bars, 100 μm. Abbreviations: NC, normal control; OE, overexpression

To evaluate the biological function of cNFIB in ICC progression, the endogenous cNFIB expression levels in 3 different ICC cell lines were firstly examined (Supplementary Fig. S2A). cNFIB was knocked down by small interfering RNAs (siRNAs) that targeting the back-splice junction in HuCCT1 and HCCC9810 cells, and was overexpressed through a lentivirus expression vector in HCCC9810 and RBE cells (Supplementary Fig. S2B). Silencing of cNFIB significantly promoted cell proliferation, cell cycle progression, cell migration and invasion of the HuCCT1 and HCCC9810 cells. Conversely, overexpression of cNFIB resulted in a considerable suppression on cell proliferation, cell cycle progression, cell migration and invasion of the HCCC9810 and RBE cells (Fig. 2A-D, Supplementary Fig. S2C-D).

**Fig. 3:**
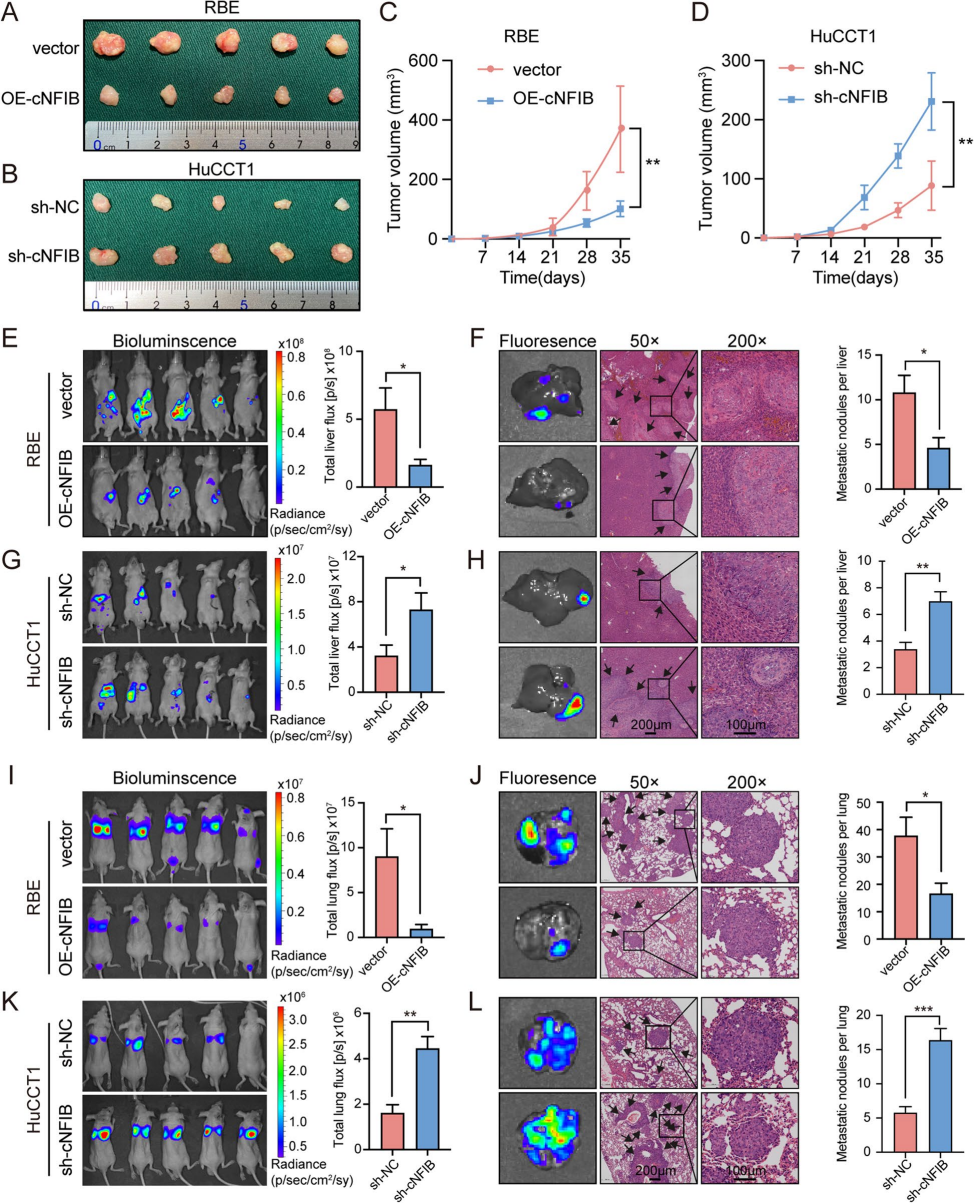
cNFIB suppresses ICC growth and metastasis in vivo. (**A**) and (**B**) Subcutaneous xenografts dissected from nude mice inoculated with the indicated cNFIB-knockdown HuCCT1 cells or cNFIB-overexpression RBE cells, *n* = 5. (**C**) and (**D**) The volume of subcutaneous xenograft tumors of indicated cells isolated from nude mice, n = 5. (**E**) and (**G**) Left, representative bioluminescent images of liver tumors from orthotopic-implantation models inoculated with cNFIB-knockdown HuCCT1 cells or cNFIB-overexpression RBE cells. Right, statistical analysis of total liver photon flux emitted from the nude mice, n = 5. (**F**) and (**H**) Representative fluorescent images (left) and hematoxylin-eosin (HE) staining of metastatic nodules (right) of liver orthotopic-implantation models inoculated with indicated RBE and HuCCT1 cells. The number of metastatic foci formed in the livers was indicated in the bar graph, n = 5. (**I**) and (**K**) Left, representative bioluminescent images of lung metastatic foci from nude mice after injection of indicated RBE and HuCCT1 cells through tail vein. Right, statistical analysis of total lung photon flux emitted from the mice, n = 5. (**J**) and (**L**) Representative fluorescent images (left) and HE staining of metastatic nodules (right) of lung metastasis models from indicated RBE and HuCCT1 cells. The number of metastatic foci formed in the lungs was indicated in the bar graph, n = 5. Data were shown as mean ± SEM, unpaired Student’s t test, *P < 0.05; **P < 0.01; ***P < 0.001

To validate these findings, we next investigated the effects of cNFIB in vivo. Stable cell lines that expressed short hairpin RNAs (sh-RNA) in HuCCT1 cells or cNFIB (OE-cNFIB) in RBE cells were established by a lentiviral transfection, which were also labeled with firefly luciferase allowing for tracking by the in vivo imaging system (IVIS). For subcutaneous implantation nude mice models, tumorigenic ability was significantly enhanced by cNFIB knockdown and dramatically inhibited after cNFIB overexpression (Fig. 3A-D and Supplementary Fig. S3A-B). Next, liver orthotopic-implantation models and lung metastasis models were established to evaluate effects of cNFIB on tumor metastasis. The IVIS imaging results suggested that cNFIB overexpression group showed smaller tumor volume and fewer metastatic foci in both liver and lung. Consistently, lower fluorescence values of GFP and less metastatic foci number of haematoxylin eosin (H&E) staining after overexpression of cNFIB confirmed its inhibitory role in tumor metastasis. In contrast, silencing of cNFIB promoted intrahepatic metastasis and lung metastasis in both models (Fig. 3E-L). Overall, above data suggested that cNFIB played an inhibitory role in ICC cell growth and metastasis in vitro and in vivo.

### cNFIB inactivates ERK signaling pathway

To dissect the molecular mechanisms of cNFIB in suppressing ICC cell proliferation and metastasis, we performed RNA-seq to identify the potential genes regulated by cNFIB knockdown. A total of 1215 genes were found to be differentially expressed, including 489 up-regulated and 726 down-regulated genes (Supplementary Fig. S4A). Kyoto Encyclopedia of Genes and Genomes (KEGG) pathway analysis showed that MAPK signaling pathway was one of the most remarkable pathways enriched, together with PI3K-Akt signaling pathway and FoxO signaling pathway. Gene Ontology (GO) analysis implied that cNFIB could regulate some important biological process (BP) involved in tumor progression, including wound healing, cell proliferation, angiogenesis, and cell adhesion (Supplementary Fig. S4B). Functional annotation revealed the genes that participated in MAPK signaling pathway, cell proliferation and cell adhesion (Supplementary Fig. S4C). The qRT-PCR and western blot results confirmed that cNFIB could regulate these selected genes associated with tumor growth and metastasis in ICC cells (Supplementary Fig. S4D-E).

**Fig. 4:**
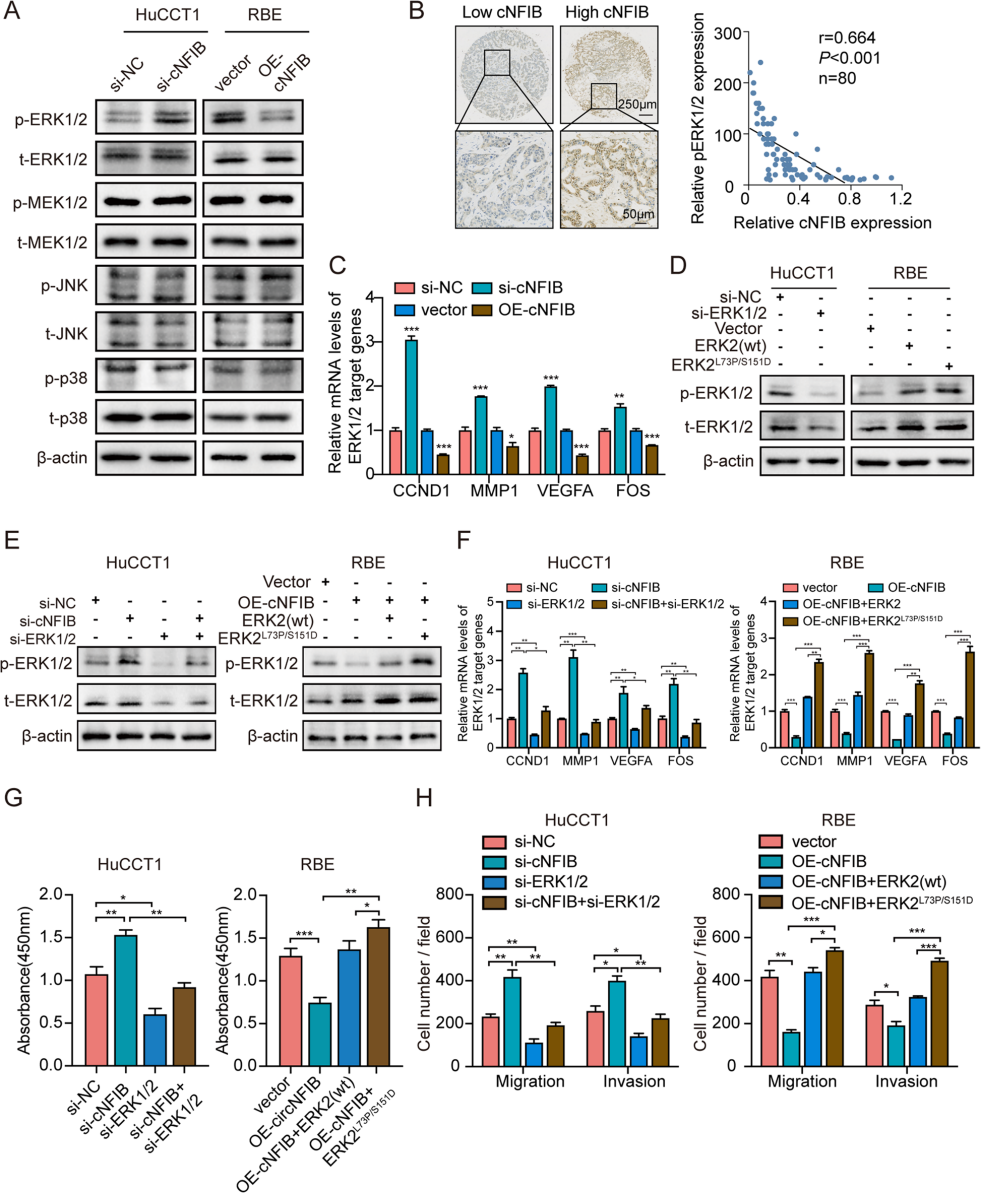
cNFIB inactivates ERK/MAPK signaling pathway. (**A**) Western blot analysis showed the expression of critical members of the MAPK signaling pathway in the indicated cells. (**B**) Left, representative images of immunohistochemical (IHC) staining of p-ERK in ICC tissues with low or high cNFIB expression. Right, the correlation between p-ERK and cNFIB levels of tumor tissues from 80 ICC patients; Pearson’s correlation test was used. (**C**) qRT-PCR analysis for the expression of downstream targets of ERK signaling in the indicated HuCCT1 (cNFIB knockdown) and RBE (cNFIB overexpression) cells. (**D**) Western blot analysis showing the expression of p-ERK and t-ERK in HuCCT1 cells transfected with ERK siRNA or RBE cells transfected with vectors expressing wild type (wt) or constitutively activated mutant ERK. (**E**) Western blot analysis showing the expression of p-ERK and t-ERK in HuCCT1 cells co-transfected with indicated siRNAs (left) and RBE cells co-transfected with indicated vectors (right). (**F**) qRT-PCR analysis for the expression of downstream targets of ERK signaling in the indicated cells. (**G**) CCK8 assays revealed cell proliferation capacity of the indicated cells co-transfected with indicated siRNAs or vectors. (**H**) Transwell assays showed the migration and invasion capacity of the indicated cells. Data were shown as mean ± SD, unpaired Student’s t test, *P < 0.05; **P < 0.01; ***P < 0.001. ERK2 (wt) was a plasmid expressing wild-type ERK2; ERK2^L73P/S151D^ was a plasmid expressing constitutively activated mutant ERK2. Abbreviations: p-ERK, phosphorylation of ERK; t-ERK, total ERK

As aberrant activation of MAPK signaling pathway is reportedly involved in oncogenesis and metastasis of various tumors [27], we postulated that cNFIB inhibited ICC growth and metastasis via regulation of MAPK signaling pathway. As was confirmed by our immunoblotting results, cNFIB knockdown in HuCCT1 cells indeed enhanced ERK phosphorylation, while overexpression of cNFIB in RBE cells caused ERK dephosphorylation. However, the phosphorylation levels of p38 and JNK were not altered after regulating cNFIB (Fig. 4A, Supplementary Fig. S5A). Notably, immunohistochemical (IHC) staining of p-ERK in ICC tissues exhibited stronger intensity in the low-cNFIB-expression group than in the high-cNFIB-expression group. The expression of cNFIB was negatively correlated with the IHC score of p-ERK in 80 ICC samples (Fig. 4B). Furthermore, we also confirmed the regulatory effects of cNFIB on CCND1, MMP1, VEGFA, and FOS expression (Fig. 4C), the key downstream targets of ERK signaling responsible for tumor proliferation and metastasis [17, 20].

We then investigated whether cNFIB deficiency-induced ICC progression was mediated by ERK phosphorylation. The phosphorylation level of ERK was decreased by ERK knockdown and increased through overexpression of wild-type ERK2 (wt) or ERK2^L73P/S151D^ (Fig. 4D), a mutant that was constitutively autophosphorylated within its activation lip [28]. As expected, knockdown of ERK could abolish the increased levels of ERK phosphorylation and its target genes caused by cNFIB depletion in HuCCT1 cells. On the other hand, reintroduction of ERK2^L73P/S151D^ restored the suppression of ERK phosphorylation, as well as the expression of ERK downstream targets, which were induced by cNFIB overexpression in RBE cells (Fig. 4E-F). Functionally, CCK-8 and transwell assays demonstrated that cNFIB inhibited cell proliferation, migration and invasion, which was dependent on and could be rescued by ERK phosphorylation (Fig. 4G-H, Supplementary Fig. S5B-C). To further validate the above results, SCH772984 was introduced to selectively block ERK phosphorylation [29]. A concentration-dependent inhibition of ERK phosphorylation was observed in HuCCT1 cells treated with SCH772984 (Supplementary Fig. S5D). cNFIB knockdown promoted ERK phosphorylation, as well as cell proliferation, migration and invasion, which was partially abrogated by SCH772984 (Supplementary Fig. S5E-G). Collectively, these data strongly indicated that loss of cNFIB facilitated ICC growth and metastasis through activation of ERK signaling pathway.

### MEK1 interacts with cNFIB and is involved in cNFIB-mediated ERK signaling inactivation and tumor suppression

**Fig. 5:**
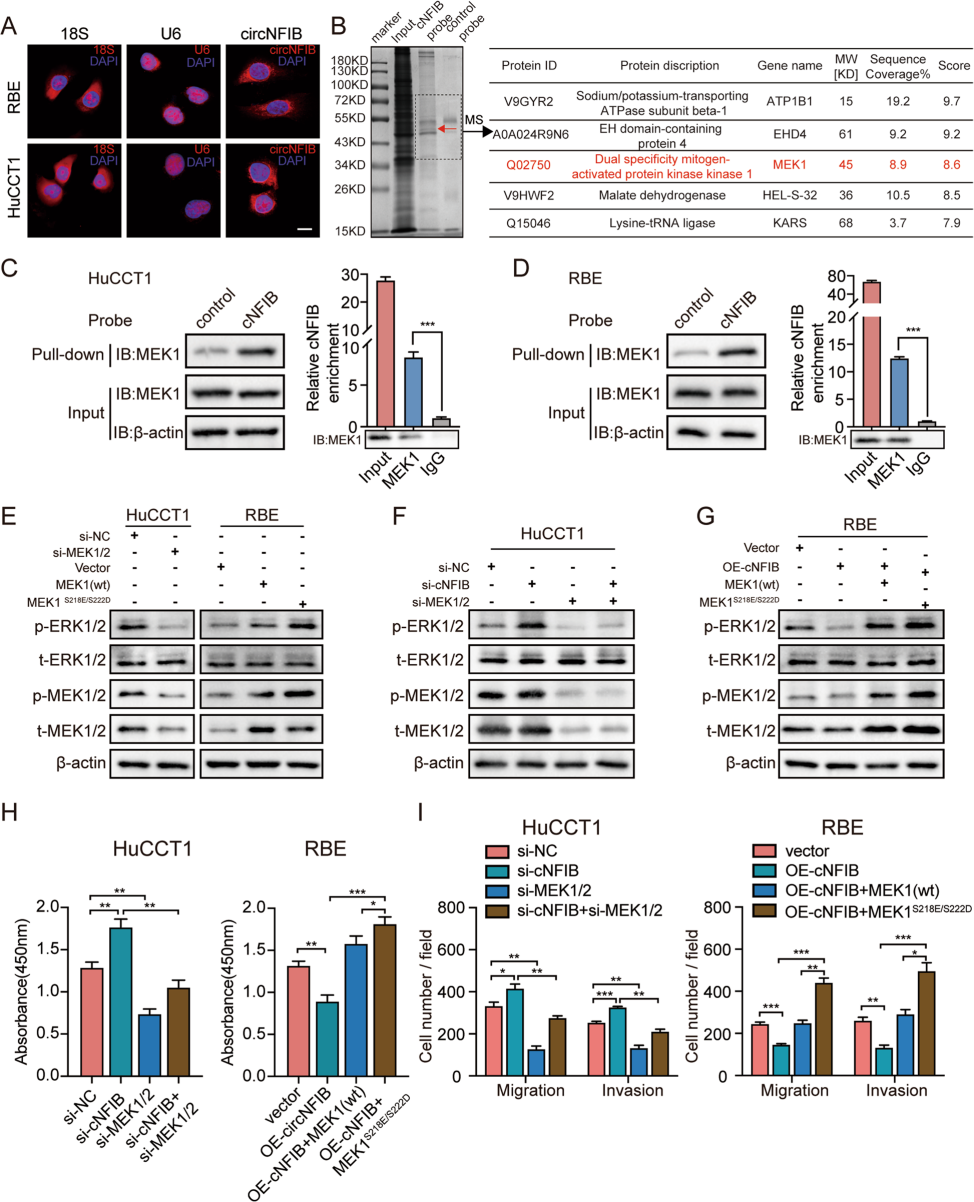
MEK1 interacts with cNFIB and is involved in cNFIB-mediated ERK signaling inactivation and tumor suppression. (**A**) Fluorescence in situ hybridization (FISH) showed cytoplasmic localization of cNFIB. Scale bars, 20 μm. (**B**) Total lysates from HuCCT1 cells were subjected to perform RNA pull-down and separated by SDS-PAGE, followed by Coomassie brilliant blue staining and mass spectrometry (MS) analysis. The specific band isolated by cNFIB probe was shown as red arrow. The table showed MS results identifying MEK1 as a potential binding protein of cNFIB. (**C**) and (**D**) The cNFIB-MEK1 interaction was validated by RNA pull-down (left) and RNA immunoprecipitation (right) assays in HuCCT1 cells (**C**) and RBE cells (**D**), respectively. (**E**) Western blot showed the expression of p-ERK, t-ERK, p-MEK and t-MEK in HuCCT1 cells transfected with MEK siRNA or RBE cells transfected with vectors expressing wild type (wt) or constitutively activated mutant MEK. (**F**) Western blot indicated the expression of p-ERK, t-ERK, p-MEK and t-MEK in HuCCT1 cells co-transfected with cNFIB siRNA and MEK siRNA. (**G**) Western blot showing the expression of p-ERK, t-ERK, p-MEK and t-MEK in in RBE cells co-transfected with indicated vectors. (**H**) The cell proliferation capacity of the indicated cells was detected by CCK8 assays. (**I**) Transwell assays showed the migration and invasion capacity in the indicated cells. Data were shown as mean ± SD, unpaired Student’s t test, *P < 0.05; **P < 0.01; ***P < 0.001. MEK1(wt) was a plasmid expressing wild-type MEK1. was MEK1^S218E/S222D^ was a plasmid expressing constitutively activated mutant MEK1. Abbreviations: p-ERK, phosphorylation of ERK; t-ERK, total ERK; p-MEK, phosphorylation of MEK; t-MEK, total MEK

To elucidate how cNFIB regulated the phosphorylation of ERK and tumor inhibition, we first performed fluorescence in situ hybridization (FISH) and RNA subcellular fractionation assays to examine the subcellular distribution of cNFIB. The results showed that cNFIB was primarily localized in the cytoplasm (Fig. 5A, Supplementary Fig. S6A). None of putative open reading frame (ORF) was annotated in circRNADb [30] implying the little protein-coding potential of cNFIB (Supplementary Fig. S6B). Additionally, RNA immunoprecipitation (RIP) showed that there was no significant difference in cNFIB enrichment between argonaute 2 (AGO2) and IgG antibody, which suggested that cNFIB unlikely functioned as a miRNA sponge (Supplementary Fig. S6C). Thus, we presumed that cNFIB might serve as a protein scaffold in the cytoplasm.

To test this hypothesis, biotinylated cNFIB probe was used to perform RNA pull-down in HuCCT1 lysates, followed by mass spectrometry (MS) analysis. We successfully enriched an array of potential binding proteins (Supplementary Table S5), and a specific protein band at approximately 45 KD was found in the Coomassie blue staining (Fig. 5B). Strikingly, MS results showed that MEK1, the primary signaling molecule in the ERK pathway, was likely to interact with cNFIB. We next conducted cNFIB pull-down and RIP assays in both HuCCT1 and RBE cells, and validated the binding between cNFIB and MEK1 (Fig. 5C-D). As MEK was the only activator of ERK phosphorylation [31], we reasoned that cNFIB might modulate ERK phosphorylation through interacting with MEK1 in ICC cells. Therefore, we first investigated the regulatory effects of MEK1 on ERK phosphorylation. Western blot data showed that knockdown of MEK significantly weakened the phosphorylation levels of MEK, as well as ERK. While, overexpression of wild-type MEK1 (wt) or MEK1^S218E/S222D^, a constitutively activated mutant [32], resulted in opposite results (Fig. 5E). As expected, MEK depletion by siRNA attenuated the promoting effects of cNFIB silencing on ERK phosphorylation and its target genes expression, whereas reintroduction of MEK1 (wt) or MEK1^S218E/S222D^ restored the cNFIB-caused reduction of ERK phosphorylation, as well as the mRNA expression levels of its downstream targets (Fig. 5F-G, Supplementary Fig. S6D). Functionally, MEK depletion weakened cell proliferation, migration and invasion that were enhanced by cNFIB knockdown. Conversely, the introduction of MEK1^S218E/S222D^ significantly erased the suppressive effects of cNFIB on cell proliferation, migration and invasion (Fig. 5H-I, Supplementary Fig. S6E-F). Furthermore, a MEK-specific inhibitor, U0126, was used to confirm the role of MEK1 on cNFIB-ERK axis. MEK phosphorylation was impeded by U0126 in a dose-dependent way (Supplementary Fig. S7A). Similarly, U0126 remarkably abolished the ERK phosphorylation, cell proliferation, migration and invasion induced by cNFIB knockdown (Supplementary Fig. S7B-D). In conclusion, our data suggested that cNFIB might inhibit ICC growth and metastasis by regulating the ERK phosphorylation via interacting with MEK1.

### cNFIB inhibits ERK phosphorylation by preventing the interaction between MEK1 and ERK2

**Fig. 6:**
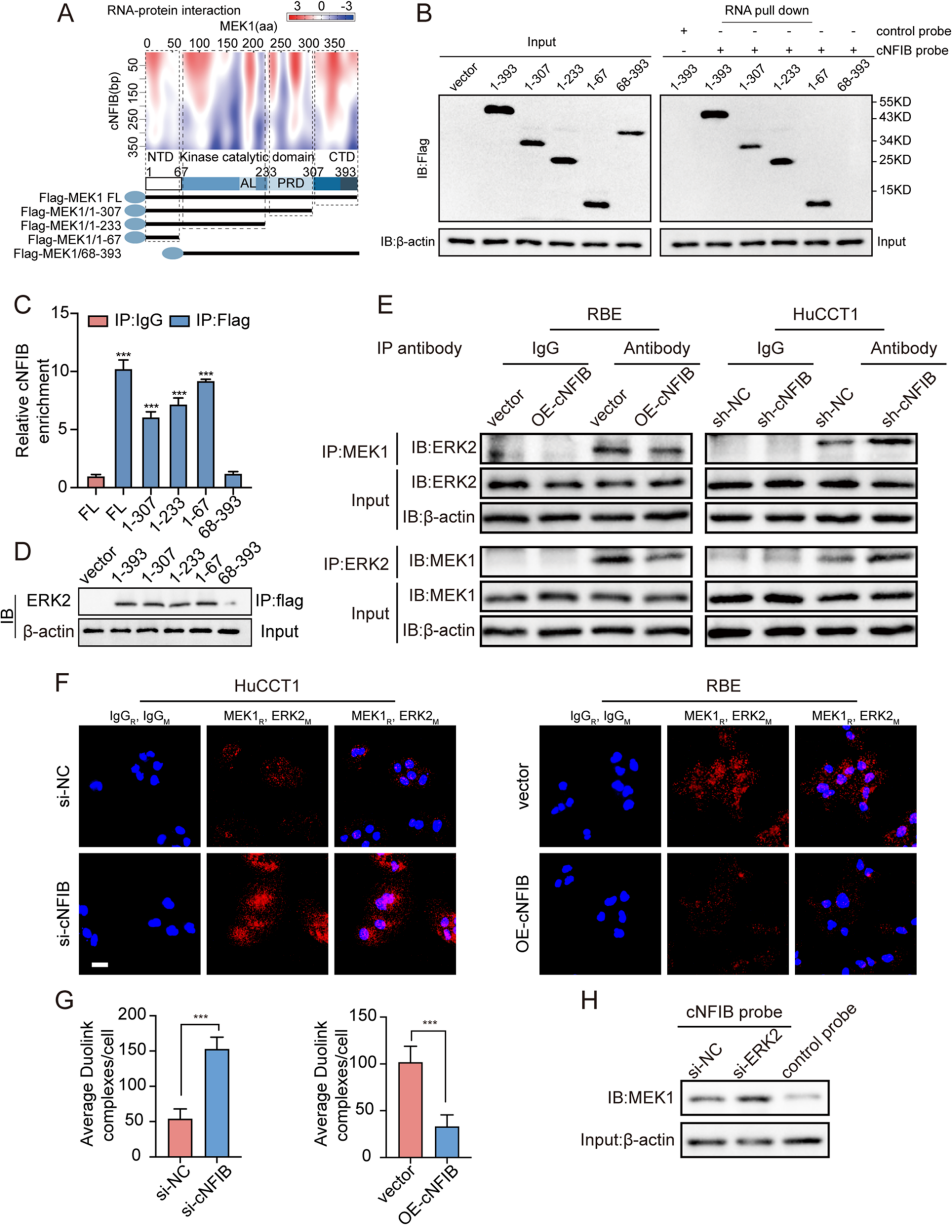
cNFIB inhibits ERK phosphorylation by preventing the interaction between MEK1 and ERK2. (**A**) Prediction of RNA-protein interaction between cNFIB and MEK1 using the catRAPID algorithm (top) and the diagrams of domain structure of MEK1 and Flag-tagged MEK1 truncations (bottom). (**B**) Left, western blot analysis showed the expression of full length (FL) or MEK1 truncations from lysates of HEK293T cells transfected with the indicated vectors; Right, western blot analysis revealed the enriched proteins by cNFIB pull-down from the lysates of HEK293T cells transfected with the indicated vectors. (**C**) RIP assays were performed in HEK293T cells transfected with the indicated vectors to validate the binding domain of MEK1 responsible for its interaction with cNFIB. (**D**) co-IP assay was conducted in HEK293T cells transfected with the indicated vectors to identify the binding domain of MEK1 responsible for its interaction with ERK2. (**E**) co-IP assay was used to examine the interaction between MEK1 and ERK2 in the RBE cells transfected with vectors expressing cNFIB (left) or HuCCT1 cells transfected with vectors expressing cNFIB shRNA (right). (**F**) Representative images of results obtained to investigate MEK1 and ERK2 interaction by Duolink in situ proximity ligation assay (PLA) assay in the indicated cells. The mouse and rabbit IgG antibodies were used as controls. Scale bars, 20 μm. (**G**) Statistical analysis of average PLA dots per cell in HuCCT1 (left) and RBE (right) cells. (**H**) Detecting the cNFIB-MEK1 complex after incubation of the biotinylated cNFIB probe with protein extracts from HuCCT1 cells transfected with the ERK2 siRNA. Data were shown as mean ± SD, unpaired Student’s t test, *P < 0.05; **P < 0.01; ***P < 0.001

Although cNFIB could bind to MEK1, there were no obvious changes in the MEK phosphorylation levels when modulating cNFIB (Fig. 4A). This prompted us to investigate whether cNFIB could affect the interaction between MEK1 and ERK. On the basis of prediction from catRAPID [33], we identified several potential domains of MEK1 responsible for binding to cNFIB (Fig. 6A, upper panel). Therefore, a series of Flag-tagged MEK1 deletion mutants were designed to analyze the interaction between MEK1 and cNFIB (Fig. 6A, lower panel). RNA pull-down and RIP assay clearly confirmed the N-terminus domain (NTD) of MEK1 (1-67aa) was essential for its interaction with cNFIB (Fig. 6B-C). A previous study demonstrated that the docking domain (D-domain) of MEK1 was responsible for its binding to ERK2. Either deletion or mutation of D-domain could decrease ERK2 phosphorylation [34]. As expected, our coimmunoprecipitation (co-IP) assay also validated ERK2 primarily bound to the NTD region of MEK1 (Fig. 6D). Interestingly, the D-domain was overlapped with NTD recognized by cNFIB, indicating that cNFIB and ERK2 might competitively bind to MEK1. Thus, we conducted co-IP to explore whether cNFIB could affect the interaction between MEK1 and ERK2. In line with our assumption, overexpression of cNFIB in RBE cells remarkably attenuated the MEK1-ERK2 interaction. In the contrast, when cNFIB was silenced, the interaction between MEK1 and ERK2 was strengthened (Fig. 6E). Moreover, Duolink in situ proximity ligation assay (PLA) also confirmed the enhanced assembly of MEK1 with ERK2 after cNFIB knockdown, and reduced interaction when cNFIB was overexpressed (Fig. 6F-G). Next, we performed cNFIB pull-down following ERK2 knockdown, and found that more MEK1 was enriched by cNFIB, further confirming competitive binding of cNFIB and ERK2 to MEK1 (Fig. 6H). In summary, these findings supported our hypothesis that cNFIB prevented the interaction between MEK1 and ERK2 by competitively binding to MEK1, thereby downregulating ERK phosphorylation and inhibiting ICC progression.

### cNFIB serves as a promising therapeutic molecule and enhances anti-tumor effects of trametinib both in vitro and in vivo

Given cNFIB suppressed ERK signaling through a different mechanism from specific MEK/ERK inhibitors, we next tested the therapeutic potential of targeting cNFIB. Both HuCCT1 and RBE cells were treated with specific inhibitor against ERK (SCH772984), and the 50% inhibitory concentration (IC50) was detected by CCK-8 assay. Notably, cNFIB overexpression significantly decreased the IC50 values of SCH772984 (Supplementary Fig. S8A). Similarly, we also found a remarkable decrease of the IC50 in U0126 (a MEK inhibitor) when cNFIB was overexpressed (Supplementary Fig. S8B). These results suggested that cells with high levels of cNFIB expression became more sensitive to small-molecule inhibitors of ERK and MEK.

Trametinib is an orally available highly specific inhibitor of MEK that had been approved for clinical treatment for advanced melanoma, anaplastic thyroid cancer and non-small-cell lung cancer [35–37]. Notably, a pre-clinical research demonstrated that dual inhibition of FGFR2 fusions and MEK (with trametinib) was considered as potential clinical utility in ICC patients [38]. Furthermore, a recent phase 2 clinical study showed the combination treatment of trametinib and dabrafenib was a promising therapeutic option in patients with BRAF^V600E^-mutated biliary tract cancer [39]. Thus, we applied trametinib to inhibit MEK and investigated the effects of cNFIB on trametinib IC50. As expected, we observed a substantial decrease of trametinib IC50 after cNFIB overexpression in both ICC cells (Supplementary Fig. S8C), indicating that cNFIB could enhance the anti-tumor activity of trametinib. Subsequently, we further evaluated the impact of cNFIB on cell growth and metastasis ability of ICC cells treated with trametinib in vitro. HuCCT1 and RBE cells were treated with trametinib for 24 h, respectively, which displayed a concentration-dependent inhibition of MEK and ERK phosphorylation (Supplementary Fig. S9A and Fig. S9E). Downregulation of cNFIB could diminish the inactivation of ERK mediated by 10 nM trametinib treatment, without affecting MEK phosphorylation level (Supplementary Fig. S9B). Accordingly, the suppression of cell proliferation, migration and invasion induced by trametinib was also attenuated after silencing cNFIB (Supplementary Fig. S9C-D). Inversely, overexpression of cNFIB could significantly further enhance the response of tumor cells to trametinib inhibition (Supplementary Fig. S9F-H).

**Fig. 7:**
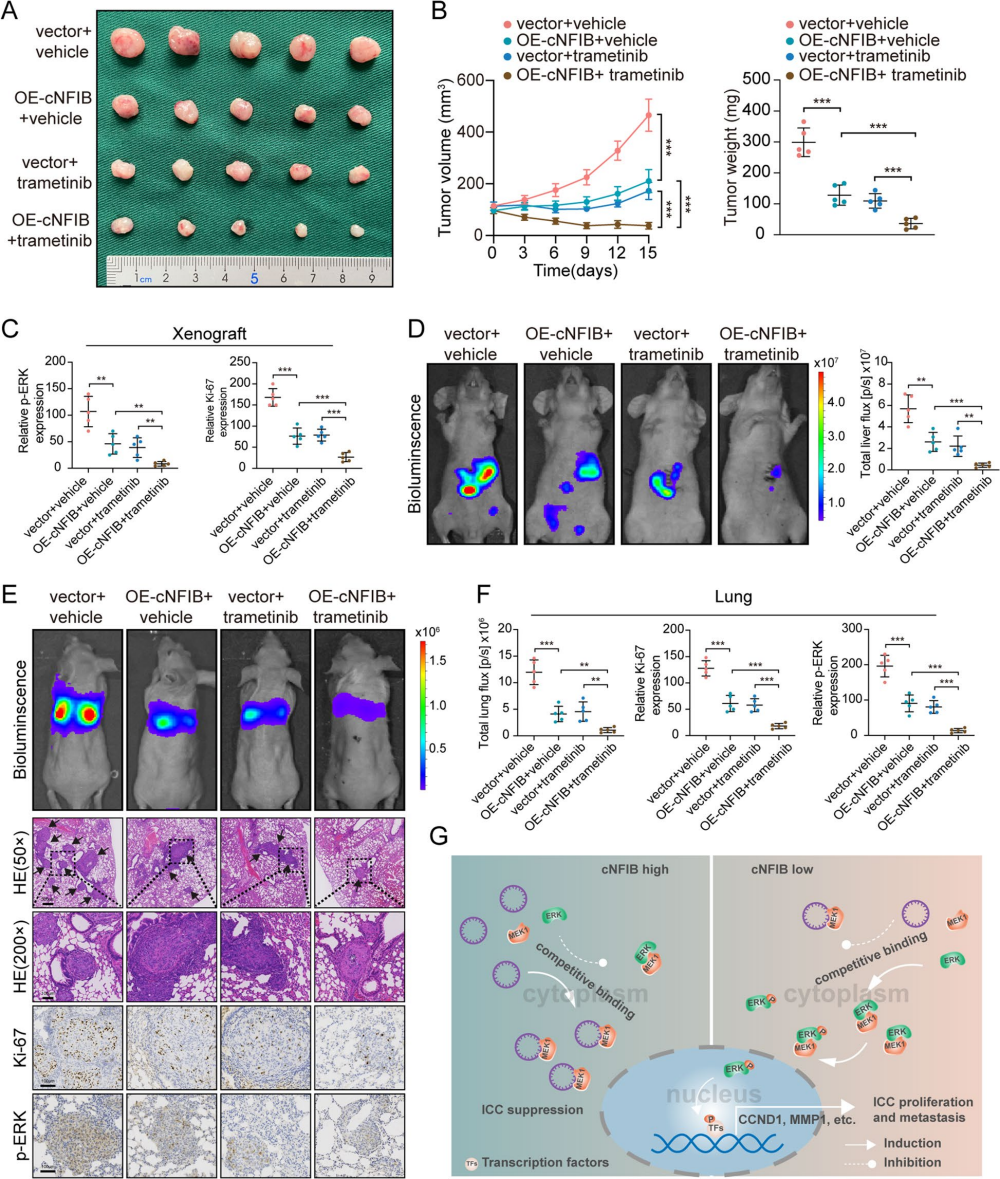
cNFIB enhances anti-tumor effects of trametinib in vivo. (**A**) Representative images of subcutaneous xenografts. RBE cells stably expressing vector or cNFIB were subcutaneously injected into the node mice. Mice were then dosed orally with or without trametinib once daily at 1 mg/kg for 15 consecutive days when subcutaneous tumor reached a volume of 100–130 mm^3^. Subcutaneous tumors were excised from node mice at day 15 after trametinib treatment. (**B**) Tumor weight and volume of subcutaneous xenografts. (**C**) Quantitative analysis of IHC staining of Ki-67 and p-ERK in xenografts. (**D**) Liver orthotopic-implantation models were established by injecting with indicated cells. Mice were then dosed orally with or without trametinib once daily at 1 mg/kg for 15 consecutive days from the 5th week after this model establishment; left, representative bioluminescent images of liver orthotopic-implantation models; right, quantitative analysis of liver photon flux emitted from the mice. (**E**) Lung metastasis models were established by injecting with indicated cells via tail vein. Mice were then dosed orally with or without trametinib once daily at 1 mg/kg for 15 consecutive days from the 7th week after this model establishment. Representative bioluminescent images of lung metastasis models (top), HE staining (middle), and of Ki-67 and p-ERK staining (bottom) of lung metastatic foci. (**F**) Quantitative analysis of total lung photon flux emitted from the mice, Ki-67 and p-ERK staining of lung metastatic lesions. (**G**) A proposed model illustrating inhibitory effects of cNFIB on ICC progression. cNFIB prevented the interaction between MEK1 and ERK by competitively binding to MEK1, thereby inactivating ERK phosphorylation. Frequent loss of cNFIB stimulated ERK phosphorylation to promote tumor cell proliferation, migration and invasion, finally inducing unfavorable prognosis of patients with ICC. Data were shown as mean ± SD, unpaired Student’s t test, *P < 0.05; **P < 0.01; ***P < 0.001

To verify the above results, we conducted in vivo experiments. RBE cells stably expressing cNFIB or vector were used to establish tumor models. Subcutaneous xenograft models revealed that upregulation of cNFIB or trametinib administration alone could suppress tumor growth, respectively, and this inhibitory effect was further strengthened by the combination of cNFIB overexpression with trametinib (Fig. 7A-B). Subsequent IHC staining of xenografts also demonstrated cNFIB upregulation plus trametinib yielded the most pervasive inhibition of ERK phosphorylation among this four groups, as well as the weakest Ki-67 staining (Fig. 7C and Supplementary Fig. S10A). In addition, similar results were observed in both liver orthotopic-implantation models and lung metastasis models. Upregulation of cNFIB in RBE cells remarkably promoted trametinib-mediated tumor suppression, as shown by less intrahepatic and lung metastasis foci compared with the other three groups (Fig. 7D-F and Supplementary Fig. S10B). Together, these data provided evidence that utilizing cNFIB to enhance the anti-tumor efficacy of trametinib might be a potential strategy in ICC therapy.

## Discussion

Although numerous improved management strategies are available in the clinic, cancer-related death of ICC remains high, which is largely caused by metastasis. With the development of sequencing techniques, a significant portion of circRNAs that are closely related to tumor metastasis have been identified. For instance, upregulation of circASAP1 in HCC contributed to tumor cell proliferation, migration, invasion, and ultimately resulted in pulmonary metastasis and poor survival of patients [9]. Loss of circCDR1as induces melanoma invasion and metastasis via interaction with IGF2BP3 [40]. However, few studies have investigated the role of circRNAs in ICC metastasis. In the present study, we identified cNFIB as a tumor suppressor that inhibited ICC growth and metastasis. We found that cNFIB was frequently deleted in metastatic ICC tissues. Importantly, cNFIB levels were inversely related to tumor numbers, TNM stage, lymph node metastasis and tumor differentiation. Patients with decreased cNFIB expression exhibited unfavorable prognosis. Moreover, gain-of-function and loss-of-function experiments suggested cNFIB negatively modulated proliferation and metastasis of ICC cells. To our knowledge, this is the first time to delineate the effects of circRNA on the metastasis of ICC, supplementing the rationale of cNIFB as a prognostic indicator for ICC patients.

Overactivation of RTK pathway is a common event during ICC development. For instance, gene fusion of FGFR2 RTK was reported to occur in around 20% ICC patients [41], and BRAF RTK mutations at the V600E locus have been identified in approximately 5% of ICC cases [42]. Therefore, aberrant alterations of RTK signaling make it amenable for therapeutic interventions at multiple levels. As one of the main pathways triggered by RTK, dysregulation of RAF/MEK/ERK signaling pathway has been identified in up to 35% of ICC [43]. Large scale studies have demonstrated that constitutive activation of ERK was associated with the pro-malignant functions of a wide number of cancers including ICC [44]. Consistent with these data, ERK signaling was hyperactive during cNFIB knockdown-induced ICC proliferation and metastasis. Blocking ERK with phosphorylation inhibitor could abolish the tumor-promoting effects of cNFIB downregulation. Generally, ERK kinase can only be activated by the upstream kinase MEK, whose role was more likely to be a “kinase gatekeeper” for ERK [31]. In our study, we demonstrated that instead of modulating the total or phosphorylation levels of MEK, cNFIB suppressed ERK phosphorylation through blocking the interaction between MEK and ERK. To sum up, our study revealed that cNFIB could serve as a key regulator of RAF/MEK/ERK signaling pathway in ICC.

Plenty of studies have been performed to understand how circRNAs exert their physiological or pathological functions. The ceRNA hypothesis is the most well-studied mechanism, proposing that circRNAs share the miRNA response elements, competitively bind to miRNAs and then regulate the expression of target genes. For example, circTP63 promoted FOXM1 expression by sponging miR-873-3p, which finally induced cell cycle progression [45]. However, this sponging hypothesis has become controversial due to the low abundance of most circRNAs in mammals, making it less likely that they could effectively exert regulatory functions via binding to miRNAs [46]. Although cNFIB was highly expressed in both ICC tissues and cell lines, it was incapable of serving as miRNA sponge because of the undetectable interaction between cNFIB and AGO2. On the other hand, mounting evidence suggests that some circRNAs can function as protein recruiters, scaffolds, and decoys in diverse biological contexts. An array of interaction patterns between circRNAs and proteins has been discovered based on more and more in-depth researches on circRNAs. After binding to proteins, circRNAs could cement or dissociate interaction between proteins, block protein from DNA or RNA, recruit proteins to chromatin, or alter protein distribution within cells [46]. Herein, we provide a potent mechanism that cNFIB can inhibit the phosphorylation of ERK by preventing the interaction between MEK1 and ERK2. Mechanistically, we found that cNIFB directly bound to the NTD region of MEK1, which contained the domain responsible for the interaction between MEK1 and ERK2 [34]. This effect impeded the binding of kinase (MEK1) to the substrate (ERK2), preventing the phosphorylation of ERK2, finally resulting in suppression of ICC proliferation and metastasis (Fig. 7G). Furthermore, administration of siRNA or inhibitor targeting MEK abrogated ERK activation and the tumor-promoting effects on ICC cells induced by cNFIB downregulation, indicating that MEK1 binding was essential for cNFIB-mediated ERK signaling regulation and ICC metastasis. An intriguing question that which binding region on cNFIB mediates the interaction between cNFIB and MEK1 requires further investigation.

ICC is an aggressive disease with limited therapeutic options. Despite advances in systemic management of patients, prognosis has not improved substantially during the past 10 years, with a 5-year survival of about 7–20% [47]. Novel treatment strategies are urgently needed to improve outcomes for patients with ICC. Recently, genetic mutations in FGFR2, IDH1 and BRAF genes have been identified in ICC, making it possible for targeted treatment [48]. For example, pemigatinib, a FGFR-specific tyrosine kinase inhibitor, has been approved by the US Food and Drug Administration (FDA) for advanced cholangiocarcinoma with FGFR2 fusions or rearrangements [49]. In addition, for patients with BRAF^V600E^-mutated biliary tract cancer, dual blockade of both BRAF (dabrafenib) and MEK (trametinib) have been considered as a promising treatment option from a phase 2, open-label, single-arm, multicenter basket trial [39]. The combination of two inhibitors provided vertical suppression of the RAF/MEK/ERK pathway, resulting in synergistic effects and stronger tumor inhibition. Similarly, one recent study also proved the synergistic effects of dual blockage. Based on the murine model mimicking FGFR2 fusion (FF)-driven ICC pathogenesis, this study demonstrated that the FF oncogenic activity in ICC required the activation of a downstream effector called MEK. BGJ398 (FF inhibitor) plus trametinib (MEK inhibitor) combination treatment generated greater therapeutic efficacy than isolated inhibitor in vitro and in vivo [38]. These results provide the notion that simultaneous inhibition of multiple molecules of an oncogenic pathway might induce stronger pathway blockage. In this regard, our data provide more evidences to support this notion. On the basis of MEK inhibition (trametinib), cNFIB competitively binds to MEK1, which results in the dissociation between MEK1 and ERK2, finally inducing more effective inhibition on ERK signaling and tumor invasion. On the other side, our finding that cNFIB is likely to generate synergistic effects on trametinib indicates that ICC cells with high levels of cNFIB might hold the potential to delay the trametinib resistance. Based on this part, we can foresee the therapeutic value of cNFIB for ICC treatment. Notably, recent advances in RNA-delivering techniques that the encapsulated circRNA SCAR was specifically delivered to the mitochondria via a nanoparticle platform for mitochondria-targeted therapy [50], raised hopes for translational application of cNFIB to treat ICC.

## Conclusions

In summary, we characterized cNFIB as a tumor suppressor to inhibit ICC proliferation and metastasis through modulating ERK signaling. Downregulation of cNFIB predicts unfavorable prognosis. Therefore, cNFIB may serve as a biomarker for patients with ICC and cNFIB-MEK-ERK axis is a potential therapeutic molecule for ICC treatment.

## Supplementary Information

**Additional file 1.**

**Additional file 2.**

**Additional file 3.**

**Additional file 4.**

**Additional file 5.**

**Additional file 6.**

**Additional file 7.**

**Additional file 8.**

**Additional file 9.**

**Additional file 10.**

**Additional file 11.**

**Additional file 12.**

**Additional file 13.**

**Additional file 14.**

**Additional file 15.**

**Additional file 16.**

**Additional file 17.**

**Additional file 18.**

**Additional file 19.**

**Additional file 20.**

## Data Availability

All data generated or analyzed during this study are included either in this article or in the supplementary information files. The raw data of circRNA-seq and RNA-seq have been deposited in the Genome Sequence Archive (GSA) in National Genomics Data Center, China National Center for Bioinformation / Beijing Institute of Genomics, Chinese Academy of Sciences (https://ngdc.cncb.ac.cn/gsa-human), Chinese Academy of Sciences, under accession numbers HRA001571 and HRA001588.

## Abbreviations

circRNAs: Circular RNAs
ICC: Intrahepatic cholangiocarcinoma
FISH: Fluorescence in situ hybridization
RIP: RNA Immunoprecipitation
MEK: Dual specificity mitogen-activated protein kinase kinase
PLA: Duolink in situ proximity ligation assay
Co-IP: Coimmunoprecipitation
ERK: Mitogen-activated protein kinase
pre-mRNA: Precursor messenger RNA
MAPK: Mitogen-activated protein kinases
JNK: The c-jun N-terminal kinase
SAPK: Stress-activated protein kinases
RTKs: Receptor tyrosine kinases
OS: Overall survival
RFS: Recurrence-free survival
TPM: Transcripts per million
qRT-PCR: Quantitative reverse transcription PCR
MVI: Microvascular invasion
siRNAs: Small interfering RNAs
sh-RNA: Short hairpin RNAs
H&E: Haematoxylin eosin
KEGG: Kyoto Encyclopedia of Genes and Genomes
GO: Gene Ontology
p-ERK: ERK phosphorylation
IHC: IMMUNOHISTOCHEMICAL
CCK-8: Cell Counting Kit-8
CCND1: Cyclin D1
MMP1: Matrix metallopeptidase 1
VEGFA: Vascular endothelial growth factor A
FOS: Fos proto-oncogene
MS: Mass spectrometry
AGO2: Argonaute 2
IC50: The 50% inhibitory concentration

## References

[1] Sung H, Ferlay J, Siegel RL, Laversanne M, Soerjomataram I, Jemal A, et al. Global Cancer Statistics 2020: GLOBOCAN estimates of incidence and mortality worldwide for 36 cancers in 185 countries. CA Cancer J Clin. 2021;(71):209–49. 10.3322/caac.21660 33538338

[2] Valle JW, Kelley RK, Nervi B, Oh DY, Zhu AX (2021). Biliary tract cancer. Lancet.

[3] Cercek A, Boerner T, Tan BR, Chou JF, Gönen M, Boucher TM, Hauser HF, Do RKG, Lowery MA, Harding JJ (2020). Assessment of hepatic arterial infusion of Floxuridine in combination with systemic gemcitabine and Oxaliplatin in patients with Unresectable intrahepatic Cholangiocarcinoma: a phase 2 clinical trial. JAMA Oncol.

[4] Doussot A, Gonen M, Wiggers JK, Groot-Koerkamp B, DeMatteo RP, Fuks D, Allen PJ, Farges O, Kingham TP, Regimbeau JM (2016). Recurrence Patterns and Disease-Free Survival after Resection of Intrahepatic Cholangiocarcinoma: Preoperative and Postoperative Prognostic Models. J Am Coll Surg.

[5] Jeck WR, Sharpless NE (2014). Detecting and characterizing circular RNAs. Nat Biotechnol.

[6] Ivanov A, Memczak S, Wyler E, Torti F, Porath HT, Orejuela MR, Piechotta M, Levanon EY, Landthaler M, Dieterich C, Rajewsky N (2015). Analysis of intron sequences reveals hallmarks of circular RNA biogenesis in animals. Cell Rep.

[7] Rybak-Wolf A, Stottmeister C, Glažar P, Jens M, Pino N, Giusti S, Hanan M, Behm M, Bartok O, Ashwal-Fluss R (2015). Circular RNAs in the mammalian brain are highly abundant, conserved, and dynamically expressed. Mol Cell.

[8] Li Q, Pan X, Zhu D, Deng Z, Jiang R, Wang X (2019). Circular RNA MAT2B promotes glycolysis and malignancy of hepatocellular carcinoma through the miR-338-3p/PKM2 Axis under hypoxic stress. Hepatology.

[9] Hu ZQ, Zhou SL, Li J, Zhou ZJ, Wang PC, Xin HY, Mao L, Luo CB, Yu SY, Huang XW (2020). Circular RNA sequencing identifies CircASAP1 as a key regulator in hepatocellular carcinoma metastasis. Hepatology.

[10] Chen RX, Chen X, Xia LP, Zhang JX, Pan ZZ, Ma XD, Han K, Chen JW, Judde JG, Deas O (2019). N(6)-methyladenosine modification of circNSUN2 facilitates cytoplasmic export and stabilizes HMGA2 to promote colorectal liver metastasis. Nat Commun.

[11] Zhang M, Zhao K, Xu X, Yang Y, Yan S, Wei P, Liu H, Xu J, Xiao F, Zhou H (2018). A peptide encoded by circular form of LINC-PINT suppresses oncogenic transcriptional elongation in glioblastoma. Nat Commun.

[12] Xu Y, Leng K, Yao Y, Kang P, Liao G, Han Y, Shi G, Ji D, Huang P, Zheng W (2021). A circular RNA, Cholangiocarcinoma-associated circular RNA 1, contributes to Cholangiocarcinoma progression, induces angiogenesis, and disrupts vascular endothelial barriers. Hepatology.

[13] Chen LL (2020). The expanding regulatory mechanisms and cellular functions of circular RNAs. Nat Rev Mol Cell Biol.

[14] Zeng Z, Xia L, Fan S, Zheng J, Qin J, Fan X, Liu Y, Tao J, Liu Y, Li K (2021). Circular RNA CircMAP3K5 acts as a MicroRNA-22-3p sponge to promote resolution of intimal hyperplasia via TET2-mediated smooth muscle cell differentiation. Circulation.

[15] Jie M, Wu Y, Gao M, Li X, Liu C, Ouyang Q, Tang Q, Shan C, Lv Y, Zhang K (2020). CircMRPS35 suppresses gastric cancer progression via recruiting KAT7 to govern histone modification. Mol Cancer.

[16] Gao X, Xia X, Li F, Zhang M, Zhou H, Wu X, Zhong J, Zhao Z, Zhao K, Liu D (2021). Circular RNA-encoded oncogenic E-cadherin variant promotes glioblastoma tumorigenicity through activation of EGFR-STAT3 signalling. Nat Cell Biol.

[17] Fang JY, Richardson BC (2005). The MAPK signalling pathways and colorectal cancer. Lancet Oncol.

[18] Cargnello M, Roux PP (2011). Activation and function of the MAPKs and their substrates, the MAPK-activated protein kinases. Microbiol Mol Biol Rev.

[19] Yoon S, Seger R (2006). The extracellular signal-regulated kinase: multiple substrates regulate diverse cellular functions. Growth Factors.

[20] Lavoie H, Gagnon J, Therrien M (2020). ERK signalling: a master regulator of cell behaviour, life and fate. Nat Rev Mol Cell Biol.

[21] von Kriegsheim A, Baiocchi D, Birtwistle M, Sumpton D, Bienvenut W, Morrice N, Yamada K, Lamond A, Kalna G, Orton R (2009). Cell fate decisions are specified by the dynamic ERK interactome. Nat Cell Biol.

[22] Sia D, Hoshida Y, Villanueva A, Roayaie S, Ferrer J, Tabak B, Peix J, Sole M, Tovar V, Alsinet C (2013). Integrative molecular analysis of intrahepatic cholangiocarcinoma reveals 2 classes that have different outcomes. Gastroenterology.

[23] Schreuer M, Jansen Y, Planken S, Chevolet I, Seremet T, Kruse V, Neyns B (2017). Combination of dabrafenib plus trametinib for BRAF and MEK inhibitor pretreated patients with advanced BRAF(V600)-mutant melanoma: an open-label, single arm, dual-Centre, phase 2 clinical trial. Lancet Oncol.

[24] Moriceau G, Hugo W, Hong A, Shi H, Kong X, Yu CC, Koya RC, Samatar AA, Khanlou N, Braun J (2015). Tunable-combinatorial mechanisms of acquired resistance limit the efficacy of BRAF/MEK cotargeting but result in melanoma drug addiction. Cancer Cell.

[25] Denny SK, Yang D, Chuang CH, Brady JJ, Lim JS, Grüner BM, Chiou SH, Schep AN, Baral J, Hamard C (2016). Nfib promotes metastasis through a widespread increase in chromatin accessibility. Cell.

[26] Zilli F, Marques Ramos P (2021). Auf der Maur P, Jehanno C, Sethi a, Coissieux MM, Eichlisberger T, Sauteur L, Rouchon a, Bonapace L, et al: the NFIB-ERO1A axis promotes breast cancer metastatic colonization of disseminated tumour cells. EMBO Mol Med.

[27] Yaeger R, Corcoran RB (2019). Targeting alterations in the RAF-MEK pathway. Cancer Discov.

[28] Emrick MA, Lee T, Starkey PJ, Mumby MC, Resing KA, Ahn NG (2006). The gatekeeper residue controls autoactivation of ERK2 via a pathway of intramolecular connectivity. Proc Natl Acad Sci U S A.

[29] Morris EJ, Jha S, Restaino CR, Dayananth P, Zhu H, Cooper A, Carr D, Deng Y, Jin W, Black S (2013). Discovery of a novel ERK inhibitor with activity in models of acquired resistance to BRAF and MEK inhibitors. Cancer Discov.

[30] Chen X, Han P, Zhou T, Guo X, Song X (2016). Li Y: circRNADb: a comprehensive database for human circular RNAs with protein-coding annotations. Sci Rep.

[31] Caunt CJ, Sale MJ, Smith PD, Cook SJ (2015). MEK1 and MEK2 inhibitors and cancer therapy: the long and winding road. Nat Rev Cancer.

[32] Mansour SJ, Matten WT, Hermann AS, Candia JM, Rong S, Fukasawa K, Vande Woude GF, Ahn NG (1994). Transformation of mammalian cells by constitutively active MAP kinase kinase. Science.

[33] Agostini F, Zanzoni A, Klus P, Marchese D, Cirillo D (2013). Tartaglia GG: catRAPID omics: a web server for large-scale prediction of protein-RNA interactions. Bioinformatics.

[34] Bromberg-White JL, Andersen NJ, Duesbery NS (2012). MEK genomics in development and disease. Brief Funct Genomics.

[35] Long GV, Hauschild A, Santinami M, Atkinson V, Mandalà M, Chiarion-Sileni V, Larkin J, Nyakas M, Dutriaux C, Haydon A (2017). Adjuvant Dabrafenib plus Trametinib in stage III BRAF-mutated melanoma. N Engl J Med.

[36] Planchard D, Smit EF, Groen HJM, Mazieres J, Besse B, Helland Å, Giannone V, D'Amelio AM, Zhang P, Mookerjee B, Johnson BE (2017). Dabrafenib plus trametinib in patients with previously untreated BRAF(V600E)-mutant metastatic non-small-cell lung cancer: an open-label, phase 2 trial. Lancet Oncol.

[37] Subbiah V, Kreitman RJ, Wainberg ZA, Cho JY, Schellens JHM, Soria JC, Wen PY, Zielinski C, Cabanillas ME, Urbanowitz G (2018). Dabrafenib and Trametinib treatment in patients with locally advanced or metastatic BRAF V600-mutant anaplastic thyroid Cancer. J Clin Oncol.

[38] Cristinziano G, Porru M, Lamberti D, Buglioni S, Rollo F, Amoreo CA, Manni I, Giannarelli D, Cristofoletti C, Russo G (2021). FGFR2 fusion proteins drive oncogenic transformation of mouse liver organoids towards cholangiocarcinoma. J Hepatol.

[39] Subbiah V, Lassen U, Élez E, Italiano A, Curigliano G, Javle M, de Braud F, Prager GW, Greil R, Stein A (2020). Dabrafenib plus trametinib in patients with BRAF(V600E)-mutated biliary tract cancer (ROAR): a phase 2, open-label, single-arm, multicentre basket trial. Lancet Oncol.

[40] Hanniford D, Ulloa-Morales A, Karz A, Berzoti-Coelho MG, Moubarak RS, Sánchez-Sendra B, Kloetgen A, Davalos V, Imig J, Wu P (2020). Epigenetic Silencing of CDR1as Drives IGF2BP3-Mediated Melanoma Invasion and Metastasis. Cancer Cell.

[41] Goyal L, Shi L, Liu LY (2019). Fece de la Cruz F, Lennerz JK, Raghavan S, Leschiner I, Elagina L, Siravegna G, Ng RWS, et al: TAS-120 overcomes resistance to ATP-competitive FGFR inhibitors in patients with FGFR2 fusion-positive intrahepatic Cholangiocarcinoma. Cancer Discov.

[42] Valle JW, Lamarca A, Goyal L, Barriuso J, Zhu AX (2017). New horizons for precision medicine in biliary tract cancers. Cancer Discov.

[43] Shroff RT, Yarchoan M, O'Connor A, Gallagher D, Zahurak ML, Rosner G, Ohaji C, Sartorius-Mergenthaler S, Parkinson R, Subbiah V (2017). The oral VEGF receptor tyrosine kinase inhibitor pazopanib in combination with the MEK inhibitor trametinib in advanced cholangiocarcinoma. Br J Cancer.

[44] Wang C, Maass T, Krupp M, Thieringer F, Strand S, Wörns MA, Barreiros AP, Galle PR, Teufel A (2009). A systems biology perspective on cholangiocellular carcinoma development: focus on MAPK-signaling and the extracellular environment. J Hepatol.

[45] Cheng Z, Yu C, Cui S, Wang H, Jin H, Wang C, Li B, Qin M, Yang C (2019). He J, et al: circTP63 functions as a ceRNA to promote lung squamous cell carcinoma progression by upregulating FOXM1. Nat Commun.

[46] Li X, Yang L, Chen LL (2018). The biogenesis, functions, and challenges of circular RNAs. Mol Cell.

[47] Zhou WY, Cai ZR, Liu J, Wang DS, Ju HQ, Xu RH (2020). Circular RNA: metabolism, functions and interactions with proteins. Mol Cancer.

[48] Banales JM, Marin JJG, Lamarca A, Rodrigues PM, Khan SA, Roberts LR, Cardinale V, Carpino G, Andersen JB, Braconi C, et al: Cholangiocarcinoma 2020: The next horizon in mechanisms and management. Nat Rev Gastroenterol Hepatol 2020, 17:557–588. 10.1038/s41575-020-0310-z PMC7447603 32606456

[49] Jain A, Javle M (2016). Molecular profiling of biliary tract cancer: a target rich disease. J Gastrointest Oncol.

[50] Abou-Alfa GK, Sahai V, Hollebecque A, Vaccaro G, Melisi D, Al-Rajabi R, Paulson AS, Borad MJ, Gallinson D, Murphy AG (2020). Pemigatinib for previously treated, locally advanced or metastatic cholangiocarcinoma: a multicentre, open-label, phase 2 study. Lancet Oncol.

