# Correction: CircNFIB inhibits tumor growth and metastasis through suppressing MEK1/ERK signaling in intrahepatic cholangiocarcinoma

**DOI:** 10.1186/s12943-023-01836-5

**Published:** 2023-08-12

**Authors:** Jinpeng Du, Tian Lan, Haotian Liao, Xuping Feng, Xing Chen, Wenwei Liao, Guimin Hou, Lin Xu, Qingbo Feng, Kunlin Xie, Mingheng Liao, Xiangzheng Chen, Jiwei Huang, Kefei Yuan, Yong Zeng

**Affiliations:** 1 Department of Liver Surgery & Liver Transplantation, State Key Laboratory of Biotherapy and Cancer Center, West China Hospital, Collaborative Innovation Center of Biotherapy, Sichuan University, Chengdu, 610041 China; 2 Laboratory of Liver Surgery, West China Hospital, Sichuan University, Chengdu, 610041 China

**Correction:** *Mol Cancer* **21**, 18 (2022)

10.1186/s12943-021-01482-9

Following publication of the original article [1], it has come to the authors’ attention that the image in Fig. 6F (the HuCCT1-sicNFIB group) appears to be doubled over itself possibly caused by an error during the microscopic image acquisition process. To avoid any confusion, we re-uploaded a clear version of this image from the same field of view. The new Fig. 6F is included below.

**Fig. 6:**
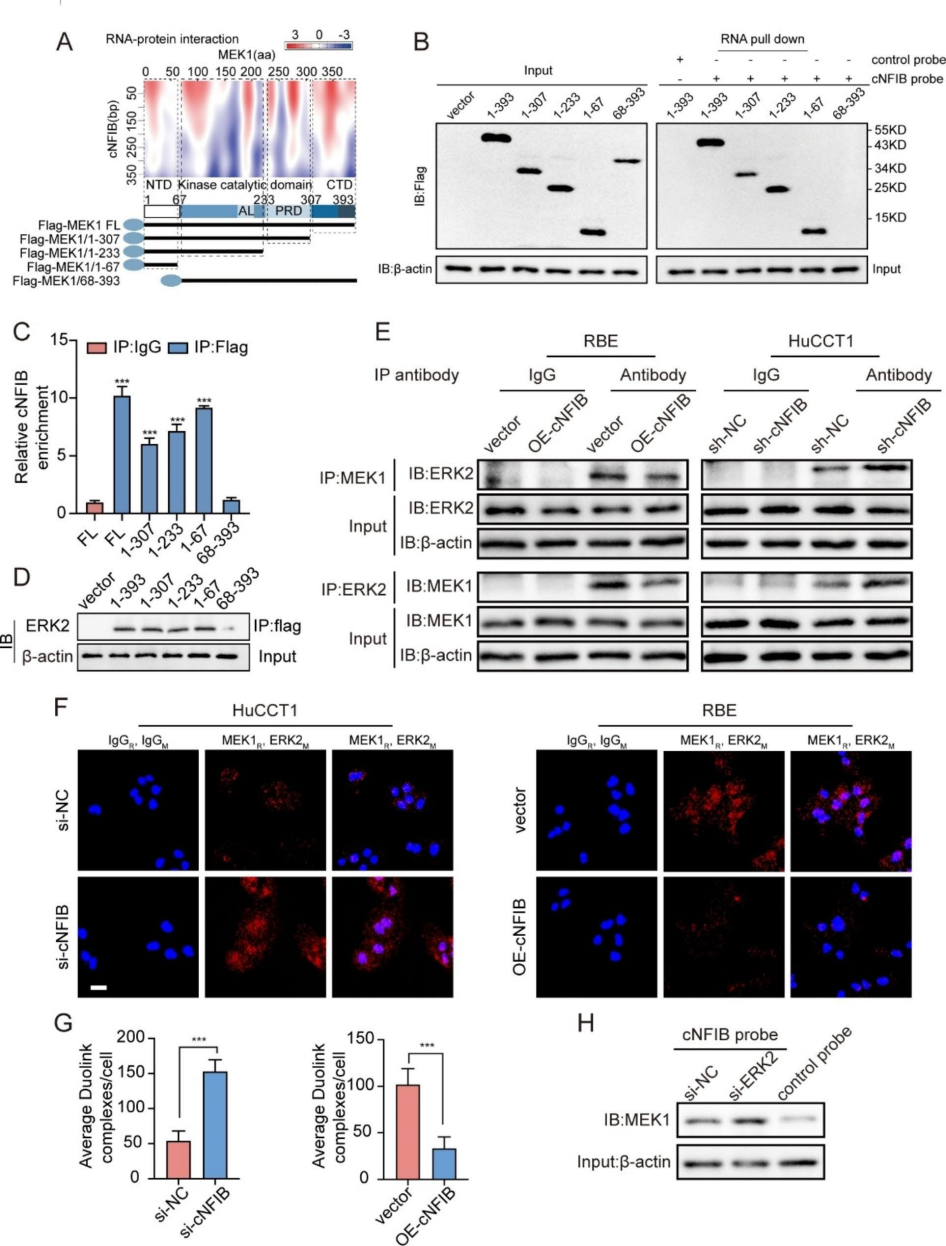
cNFIB inhibits ERK phosphoryla tion by preventing the interaction between MEK1 and ERK2. **(A)** Prediction of RNA-protein interaction between cNFIB and MEK1 using the catRAPID algorithm (top) and the diagrams of domain structure of MEK1 and Flag-tagged MEK1 truncations (bottom). **(B)** Left, western blot analysis showed the expression of full length (FL) or MEK1 truncations from lysates of HEK293T cells transfected with the indicated vectors; Right, western blot analysis revealed the enriched proteins by cNFIB pull-down from the lysates of HEK293T cells transfected with the indicated vectors. **(C)** RIP assays were performed in HEK293T cells transfected with the indicated vectors to validate the binding domain of MEK1 responsible for its interaction with cNFIB. **(D)** co-IP assay was conducted in HEK293T cells transfected with the indicated vectors to identify the binding domain of MEK1 responsible for its interaction with ERK2. **(E)** co-IP assay was used to examine the interaction between MEK1 and ERK2 in the RBE cells transfected with vectors expressing cNFIB (left) or HuCCT1 cells transfected with vectors expressing cNFIB shRNA (right). **(F)** Representative images of results obtained to investigate MEK1 and ERK2 interaction by Duolink in situ proximity ligation assay (PLA) assay in the indicated cells. The mouse and rabbit IgG antibodies were used as controls. Scale bars, 20 μm. **(G)** Statistical analysis of average PLA dots per cell in HuCCT1 (left) and RBE (right) cells. **(H)** Detecting the cNFIB-MEK1 complex after incubation of the biotinylated cNFIB probe with protein extracts from HuCCT1 cells transfected with the ERK2 siRNA. Data were shown as mean ± SD, unpaired Student’s t test, *P < 0.05; **P < 0.01; ***P < 0.001.
