# Supplementary material for: CircNFIB inhibits tumor growth and metastasis through suppressing MEK1/ERK signaling in intrahepatic cholangiocarcinoma
Source: Mol Cancer. 2022 Jan 17;21:18. doi: 10.1186/s12943-021-01482-9 (PMC8762882; doi:10.1186/s12943-021-01482-9)
Supplement: Supplementary file 1 — Additional file 1. [file 12943_2021_1482_MOESM1_ESM.docx]

| Clinical characteristics | No metastasis  (n=15) | Metastasis  (n=15) | P-value |
| --- | --- | --- | --- |
| Age, year, >60/≤60 | 7/8 | 5/10 | 0.7104 |
| Gender, male/female | 7/8 | 9/6 | 0.7152 |
| Ascites, present/absent | 1/14 | 4/11 | 0.3295 |
| Hepatolithiasis, present/absent | 0/15 | 1/14 | >0.9999 |
| HbsAg, positive/negative | 2/13 | 4/11 | 0.6513 |
| CA19-9, >22/≤22 | 8/7 | 10/5 | 0.7104 |
| Tumor size (cm), >5/≤5 | 9/6 | 12/3 | 0.4270 |
| Tumor number, multiple/solitary | 1/14 | 3/12 | 0.5977 |
| Differentiation, poor/well-moderate | 10/5 | 13/2 | 0.3898 |
| MVI, present/absent | 0/15 | 2/13 | 0.4828 |
| Lymph node, positive/negative | 0/15 | 2/13 | 0.4828 |
| Cirrhosis, with/without | 0/15 | 2/13 | 0.4828 |
| TNM Stage, III/I- II | 12/3 | 12/3 | >0.9999 |

**Table S1. Clinical characteristics of 30 ICC patients used for circRNA-seq**

ICC, intrahepatic cholangiocarcinoma; MVI, microvascular invasion; TNM, tumor-node-metastasis;
