# Supplementary material for: CircNFIB inhibits tumor growth and metastasis through suppressing MEK1/ERK signaling in intrahepatic cholangiocarcinoma
Source: Mol Cancer. 2022 Jan 17;21:18. doi: 10.1186/s12943-021-01482-9 (PMC8762882; doi:10.1186/s12943-021-01482-9)
Supplement: Supplementary file 2 — Additional file 2. [file 12943_2021_1482_MOESM2_ESM.docx]

| Variables | Low cNFIB  (n = 57) | High cNFIB  (n = 57) | *P* value |
| --- | --- | --- | --- |
| Age, year, >60/≤60 | 26/31 | 24/33 | 0.7058 |
| Gender, male/female  Ascites, present/absent | 24/33  5/52 | 30/27  5/52 | 0.2604  >0.999 |
| Hepatolithiasis, present/absent | 2/55 | 1/56 | 0.5585 |
| HbsAg, positive/negative | 9/48 | 13/44 | 0.3425 |
| CA19-9, >22/≤22 | 37/20 | 39/18 | 0.6911 |
| Tumor size (cm) >5/≤5 | 36/21 | 35/22 | 0.8468 |
| Tumor number, multiple/solitary | 16/41 | 15/42 | 0.8333 |
| Differentiation, poor/well-moderate | 41/16 | 37/20 | 0.4303 |
| MVI, present/absent | 5/52 | 7/50 | 0.5416 |
| Lymph node, positive/negative | 8/49 | 8/49 | >0.999 |
| Cirrhosis, with/without | 6/51 | 6/51 | >0.999 |
| TNM stage, III/I- II | 37/20 | 36/21 | 0.8453 |

**Table S2. Clinical characteristics of ICC patients (Propensity Score Matched) based on cNFIB expression levels.**

ICC, intrahepatic cholangiocarcinoma; MVI, microvascular invasion; TNM, tumor-node-metastasis;
