# Supplementary material for: CircNFIB inhibits tumor growth and metastasis through suppressing MEK1/ERK signaling in intrahepatic cholangiocarcinoma
Source: Mol Cancer. 2022 Jan 17;21:18. doi: 10.1186/s12943-021-01482-9 (PMC8762882; doi:10.1186/s12943-021-01482-9)
Supplement: Supplementary file 3 — Additional file 3. [file 12943_2021_1482_MOESM3_ESM.docx]

**Table S3. Univariate analysis of several variables for OS and RFS of the matched cohort.**

| Variables | Overall survival | | Recurrence-free survival | |
| --- | --- | --- | --- | --- |
|  | Hazzard ratio (95% CI) | *P* value | Hazzard ratio (95% CI) | *P* value |
| Age, year, >60/≤60 | 1.029 (0.611-1.733) | 0.913 | 0.940 (0.617-1.433) | 0.774 |
| Gender, male/female | 1.071 (0.635-1.806) | 0.798 | 0.930 (0.610-1.418) | 0.736 |
| Ascites, present/absent | 1.226 (0.486-3.094) | 0.626 | 1.709 (0.847-3.4468) | 0.134 |
| Hepatolithiasis, present/absent | 0.861 (0.206-3.601) | 0.837 | 1.277 (0.402-4.058) | 0.679 |
| HbsAg, positive/negative | 1.277 (0.641-2.545) | 0.487 | 1.312 (0.771-2.233) | 0.317 |
| CA19-9, >22/≤22 | 1.040 (0.589 -1.837) | 0.892 | 0.946 (0.610-1.467) | 0.804 |
| Tumor size (cm) >5/≤5 | 1.617 (0.919-2.847) | 0.096 | 1.794 (1.134-2.838) | **0.012** |
| Tumor number, multiple/solitary | 2.876 (1.585-5.218) | **0.001** | 2.212 (1.369-3.575) | **0.001** |
| Differentiation, poor/well-moderate | 2.448 (1.281-4.680) | **0.007** | 1.965 (1.203-3.210) | **0.007** |
| MVI, present/absent | 1.956 (0.918-4.169) | 0.082 | 1.603 (0.824-3.118) | 0.164 |
| Lymph node, positive/negative | 2.617 (1.265-5.412) | **0.009** | 1.731 (0.938-3.192) | 0.079 |
| Cirrhosis, with/without | 1.751 (0.879-3.485) | 0.111 | 1.264 (0.661-2.350) | 0.479 |
| TNM stage, III/I- II | 2.534 (1.409-4.558) | **0.002** | 1.587 (1.019-2.471) | **0.041** |
| cNFIB expression, low/high | 2.001 (1.175-3.407) | **0.011** | 1.663 (1.089-2.539) | **0.019** |

MVI, microvascular invasion; CI, confidence interval; TNM, tumor-node-metastasis.
