# Supplementary material for: CircNFIB inhibits tumor growth and metastasis through suppressing MEK1/ERK signaling in intrahepatic cholangiocarcinoma
Source: Mol Cancer. 2022 Jan 17;21:18. doi: 10.1186/s12943-021-01482-9 (PMC8762882; doi:10.1186/s12943-021-01482-9)
Supplement: Supplementary file 4 — Additional file 4. [file 12943_2021_1482_MOESM4_ESM.docx]

**Table S4. Multivariate analysis of several variables for OS and RFS of the matched cohort.**

| Variables | Overall survival | | Recurrence-free survival | |
| --- | --- | --- | --- | --- |
|  | Hazzard ratio (95% CI) | *P* value | Hazzard ratio (95% CI) | *P* value |
| Tumor size (cm) >5/≤5 | - | ns | - | ns |
| Tumor number, multiple/solitary | 2.118 (1.142-3.928) | 0.017 | 1.808 (1.100-2.972) | 0.019 |
| Differentiation, poor/well-moderate | 2.094 (1.067-4.108) | 0.032 | 1.667 (1.009-2.753) | 0.046 |
| MVI, present/absent | - | ns | - | ns |
| Lymph node, positive/negative | - | ns | - | ns |
| TNM stage, III/I- II | 2.196 (1.138-4.236) | 0.019 | - | ns |
| cNFIB expression, low/high | 2.185 (1.247-3.828) | 0.006 | 1.634 (1.059-2.521) | 0.026 |

MVI, microvascular invasion; CI, confidence interval; TNM, tumor-node-metastasis; ns, no significance.
