# Supplementary material for: CircNFIB inhibits tumor growth and metastasis through suppressing MEK1/ERK signaling in intrahepatic cholangiocarcinoma
Source: Mol Cancer. 2022 Jan 17;21:18. doi: 10.1186/s12943-021-01482-9 (PMC8762882; doi:10.1186/s12943-021-01482-9)
Supplement: Supplementary file 6 — Additional file 6. [file 12943_2021_1482_MOESM6_ESM.docx]

| Name | Forward sequence | Reverse sequence | Supplier |
| --- | --- | --- | --- |
| β-actin | CTCGCCTTTGCCGATCC | TCTCCATGTCGTCCCAGTTG | TsingKe |
| U6 | TCGGCAGCACATATACTAAAATTGG | ACGAATTTGCGTGTCATCCT | TsingKe |
| U3 | TTCTCTGAGCGTGTAGAGCACCGA | GATCATCAATGGCTGACGGCAGTT | TsingKe |
| GAPDH | GAAGGTGAAGGTCGGAGTCA | TTGAGGTCAATGAAGGGGTC | TsingKe |
| mNFIB | TTTGTGTCCAGCCACATCAT | GTGGCTTGGACTTCCTGATT | TsingKe |
| Pre-mNFIB | GCTAGGGGCTACTATATTGGGC | AAGGGGTCCTGTACACTCGA | TsingKe |
| cNFIB | CGAAAGAGATCAAGATTCTGGAC | CCTGGGTTATGGGCGTTCT | TsingKe |
| cZNF215 | TCAGATGCCTGGAAAGATATGC | CCCCACTCTTCCTTGCTGAA | TsingKe |
| cCD109 | TCAAGTTCAAGTGAATGCTCTTTT | TGGTCTCAAATGATAAGCGGGT | TsingKe |
| cPLOD2 | TGGAAATGGACCCACCAAGA | CCTTGACCAAGGACCTTCACA | TsingKe |
| cBNIP3L | CTTTGGGGCTAGGGTTCCTG | TCTCCATGTCTCCATTGTGGA | TsingKe |
| cUBE2D2 | TCTACGATCACAGTGGTCTCC | CGTGCCAGATCATTCAATTCTTTTCT | TsingKe |
| cAKT3 | AATGGGGGCGAGGAGAATAT | GTTTGGCTTTGGTCGTTCTGT | TsingKe |
| cCRIM1 | AGAGAATTGAAGCTGCGGGG | CCGCCTTACGTTAGAGCTGT | TsingKe |
| cCRIM1-2 | GAGGTACTACGTGCCCGAAG | TCCAGTTCTCATCTTCGCACA | TsingKe |
| VEGFA | AAGCGCAAGAAATCCCGGTA | CGCGAGTCTGTGTTTTTGCA | TsingKe |
| FOS | GGGAGGACCTTATCTGTGCG | ACACACTCCATGCGTTTTGC | TsingKe |
| CCND1 | CTGATTGGACAGGCATGGGT | TCTTGCCACCTCCCTTCAAC | TsingKe |
| MMP1 | TGTGGTGTCTCACAGCTTCC | CAACTTGCCTCCCATCATTCT | TsingKe |

**Table S6. Primers used in this study.**
