# Supplementary material for: CircNFIB inhibits tumor growth and metastasis through suppressing MEK1/ERK signaling in intrahepatic cholangiocarcinoma
Source: Mol Cancer. 2022 Jan 17;21:18. doi: 10.1186/s12943-021-01482-9 (PMC8762882; doi:10.1186/s12943-021-01482-9)
Supplement: Supplementary file 7 — Additional file 7. [file 12943_2021_1482_MOESM7_ESM.docx]

| Name | Target sequence | Supplier |
| --- | --- | --- |
| si-cNFIB | AAGAGATCAAGATTCTGGA | Ribobio |
| si-ERK | GCAATGACCATATCTGCTA | Ribobio |
| si-MEK | CCAGTGGAGTGTTCAGTCT | Ribobio |

**Table S7. The target sequences of siRNAs used in this study.**
