# Supplementary material for: CircNFIB inhibits tumor growth and metastasis through suppressing MEK1/ERK signaling in intrahepatic cholangiocarcinoma
Source: Mol Cancer. 2022 Jan 17;21:18. doi: 10.1186/s12943-021-01482-9 (PMC8762882; doi:10.1186/s12943-021-01482-9)
Supplement: Supplementary file 8 — Additional file 8. [file 12943_2021_1482_MOESM8_ESM.docx]

| Name | Target sequence | Supplier |
| --- | --- | --- |
| cNFIB biotinylated probe-1 | CAGAATCTTGATCTCTTTCG-/3bio/ | Ribobio |
| cNFIB biotinylated probe-2 | TTGTCCAGAATCTTGATCTC-/3bio/ | Ribobio |
| Control biotinylated probe | CTGCGTCATAATTTCGTCTA-/3bio/ | Ribobio |

**Table S8. Biotinylated probes used in this study.**
