# Supplementary material for: CircNFIB inhibits tumor growth and metastasis through suppressing MEK1/ERK signaling in intrahepatic cholangiocarcinoma
Source: Mol Cancer. 2022 Jan 17;21:18. doi: 10.1186/s12943-021-01482-9 (PMC8762882; doi:10.1186/s12943-021-01482-9)
Supplement: Supplementary file 9 — Additional file 9. [file 12943_2021_1482_MOESM9_ESM.docx]

**Table S9. Antibodies and reagents used in this study.**

| Name | Supplier | Cat no. |
| --- | --- | --- |
| Anti-ERK1/2 Mouse mAb | Proteintech | 67170-1-Ig |
| Anti-Phospho ERK1(T202/Y204) + ERK2 (T185/Y187) Rabbit pAb | ABclonal | AP0472 |
| Anti-MEK1/2 Rabbit mAb | ABclonal | A4868 |
| Anti- Phospho MEK1(S217) + MEK2 (S221) Rabbit pAb | ABclonal | AP0209 |
| Anti-MEK1 Rabbit mAb | abcam | ab32576 |
| Anti-ERK2 Rabbit pAb | abcam | ab227134 |
| Anti-ERK2 Mouse mAb | Affinity | BF0298 |
| Anti-JNK Rabbit pAb | CST | 9252T |
| Anti- Phospho JNK (Thr183/Tyr185) Mouse mAb | CST | 9255S |
| Anti-p38 Rabbit pAb | CST | 9212S |
| Anti- Phospho p38 (Thr180/Tyr182) Mouse mAb | CST | 9216s |
| Anti-β-actin Mouse mAb | ZEN-bioscience | 200068-8F10 |
| Rabbit IgG Isotype Control | ThermoFisher SCIENTIFIC | 02-6102 |
| Mouse IgG Isotype Control | ThermoFisher SCIENTIFIC | 10400C |
| Anti-Flag Rabbit mAb | CST | 14793S |
| Goat anti-Mouse IgG (H&L)（HRP conjugate） | ZEN-bioscience | 511103 |
| Goat anti-Rabbit IgG (H&L)（HRP conjugate） | ZEN-bioscience | 511203 |
| SCH772984 | Selleck | S7101 |
| U0126 | MCE | HY-12031 |
| Trametinib | aladdin | 871700-17-3 |
