# Supplementary figures and images for: CircNFIB inhibits tumor growth and metastasis through suppressing MEK1/ERK signaling in intrahepatic cholangiocarcinoma
Source: Mol Cancer. 2022 Jan 17;21:18. doi: 10.1186/s12943-021-01482-9 (PMC8762882; doi:10.1186/s12943-021-01482-9)

**Figure S1****A**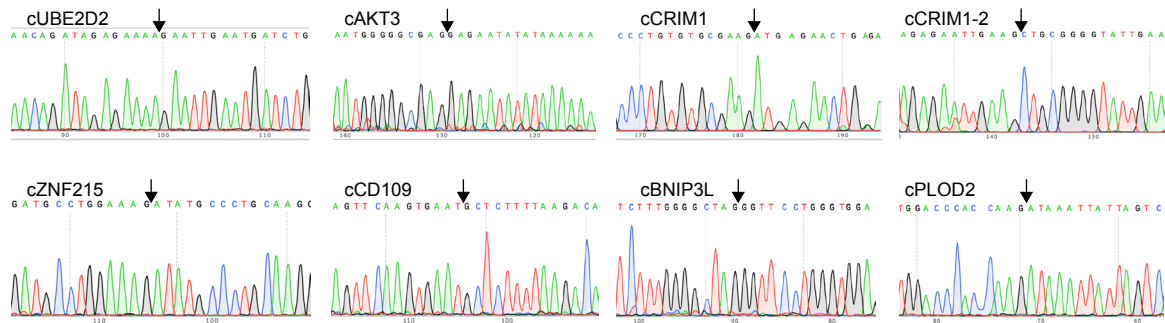**B**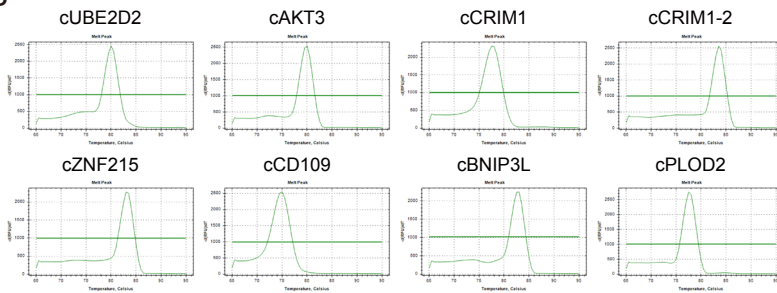**C**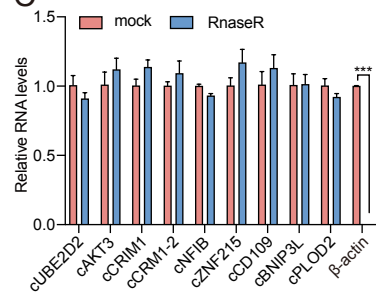**D**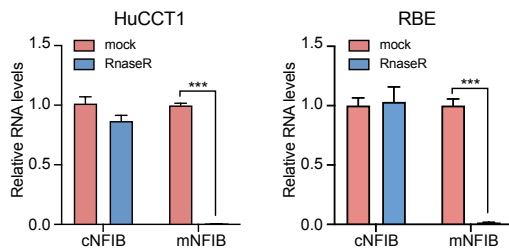**E**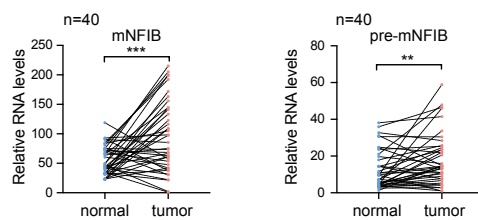**F**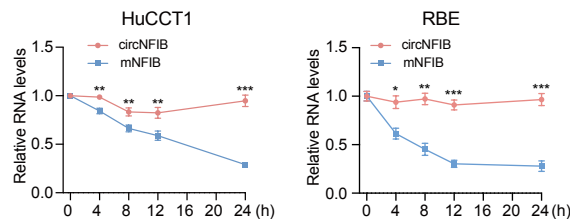**G**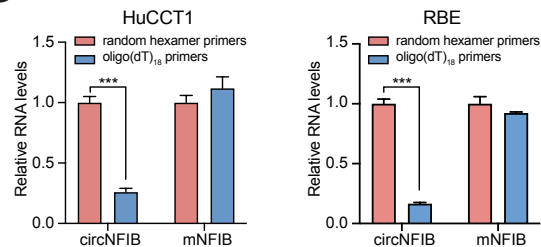

Supplement: Supplementary file 10 — Additional file 10. [file 12943_2021_1482_MOESM10_ESM.pdf]

**Figure S2****A**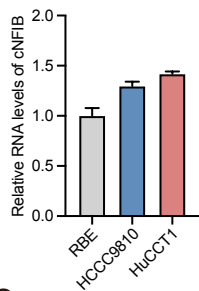**B**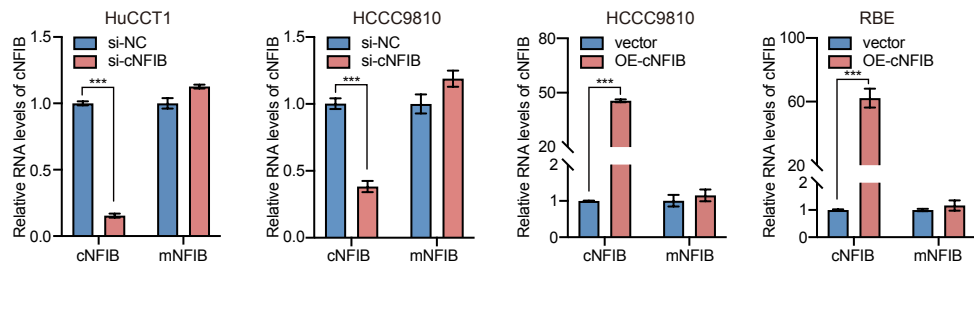**C**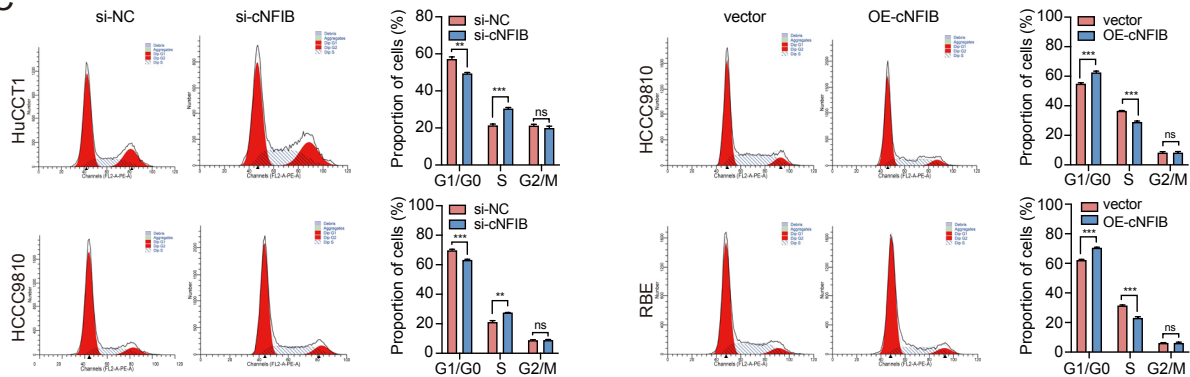**D**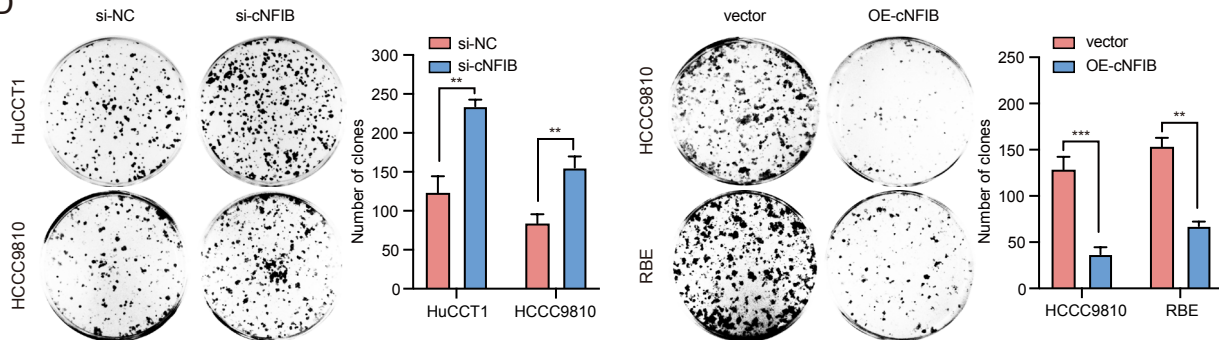

Supplement: Supplementary file 11 — Additional file 11. [file 12943_2021_1482_MOESM11_ESM.pdf]

# Figure S3

A

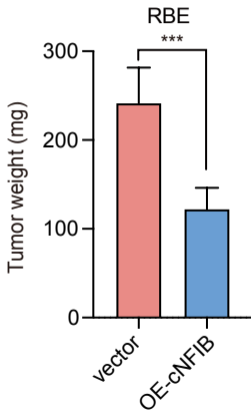

B

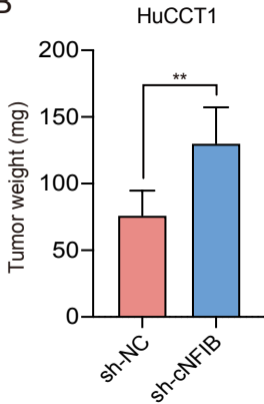

Supplement: Supplementary file 12 — Additional file 12. [file 12943_2021_1482_MOESM12_ESM.pdf]

**Figure S4**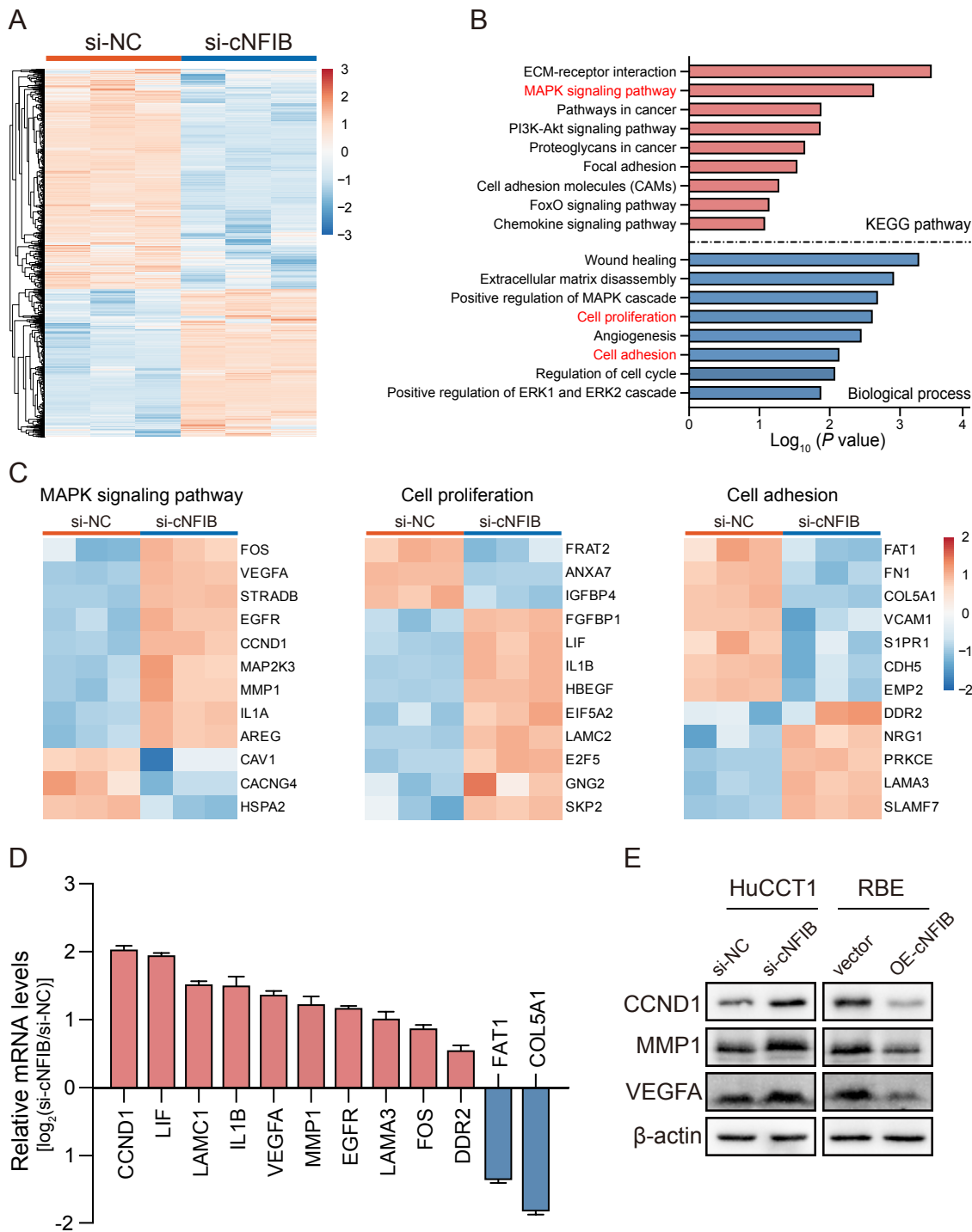

Supplement: Supplementary file 13 — Additional file 13. [file 12943_2021_1482_MOESM13_ESM.pdf]

**Figure S5**

**A**

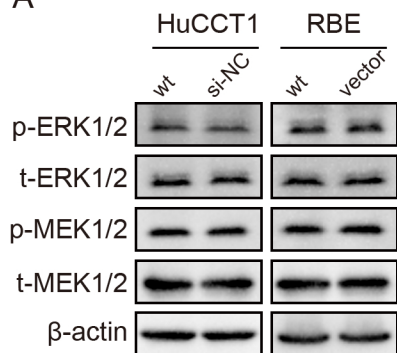

**B**

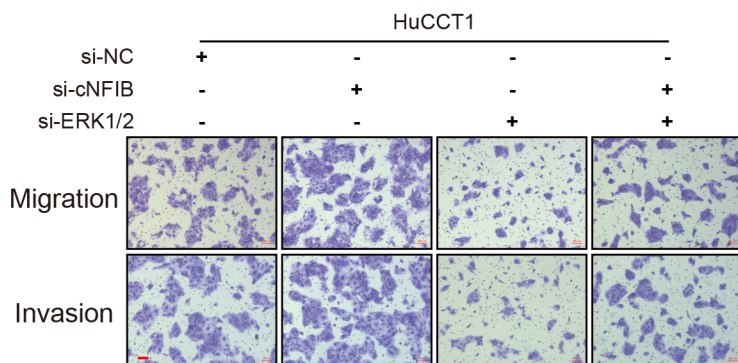

**C**

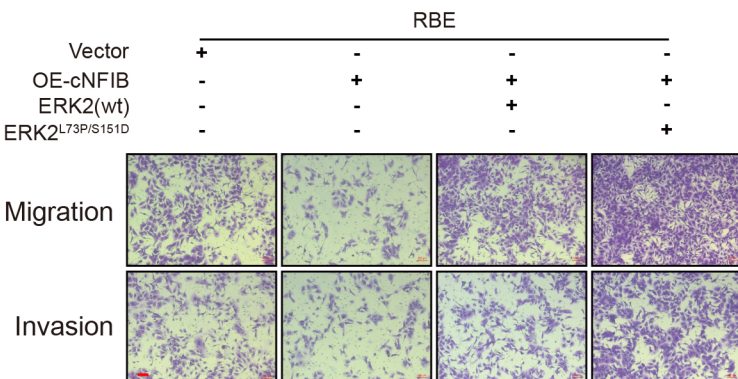

**D**

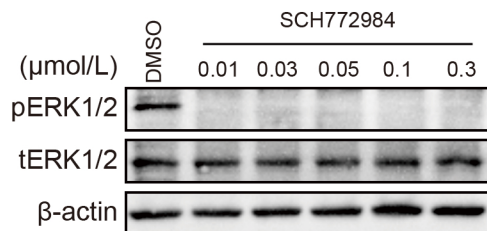

**E**

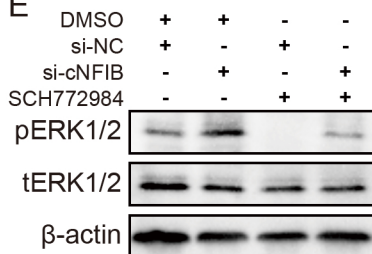

**F**

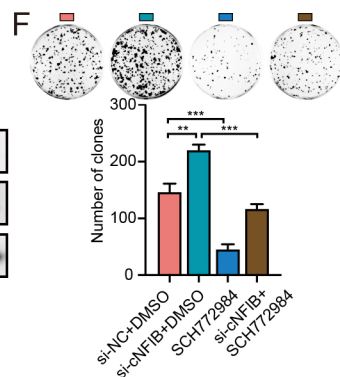

**G**

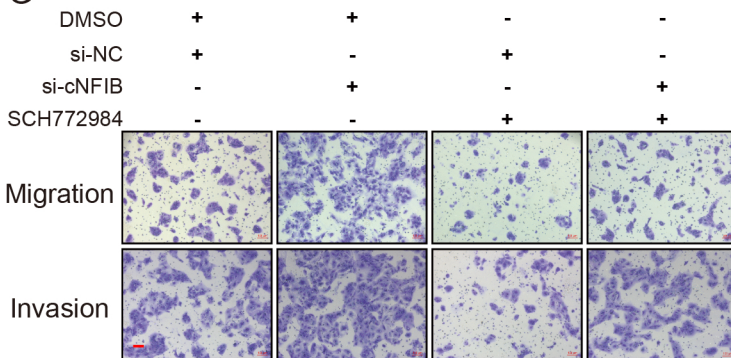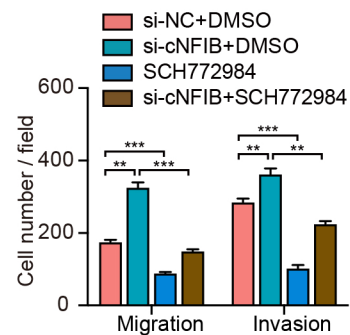

Supplement: Supplementary file 14 — Additional file 14. [file 12943_2021_1482_MOESM14_ESM.pdf]

**Figure S7****A**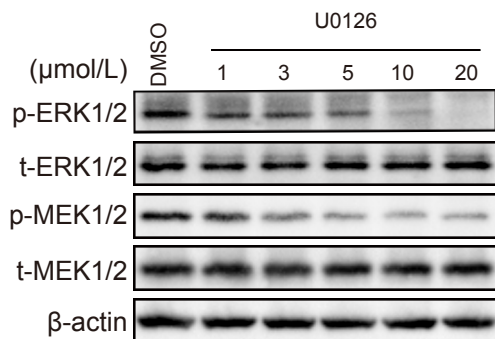**B**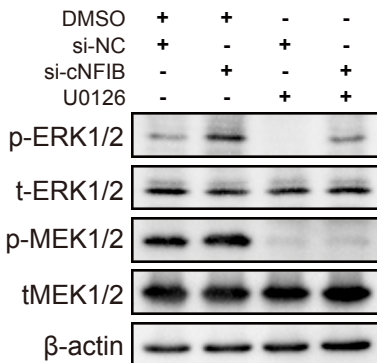**C**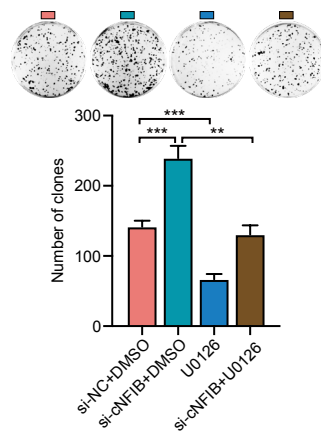**D**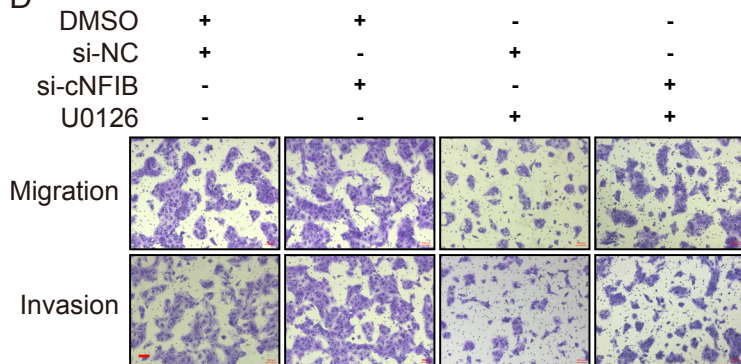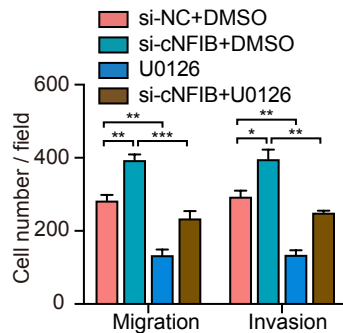

Supplement: Supplementary file 16 — Additional file 16. [file 12943_2021_1482_MOESM16_ESM.pdf]

**Figure S8**

**A**

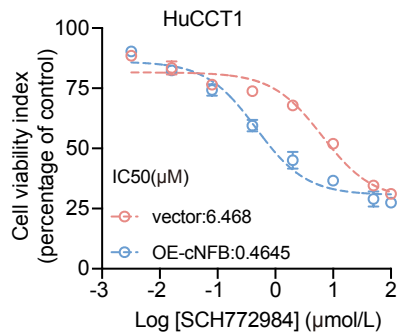

**B**

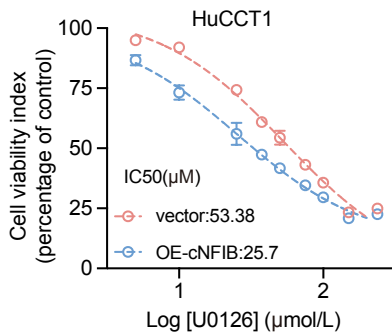

**C**

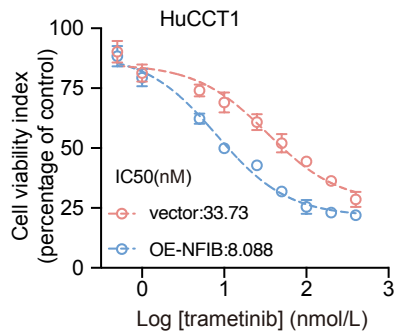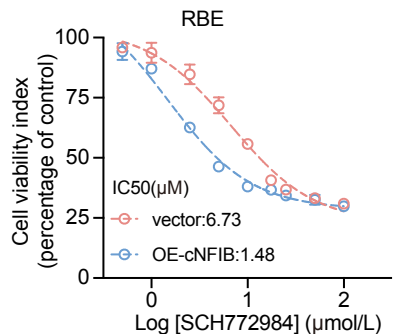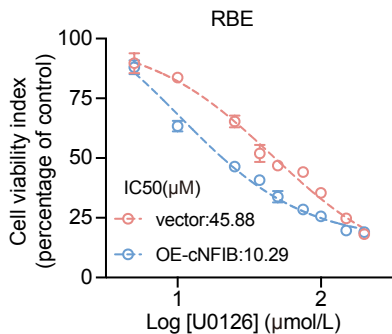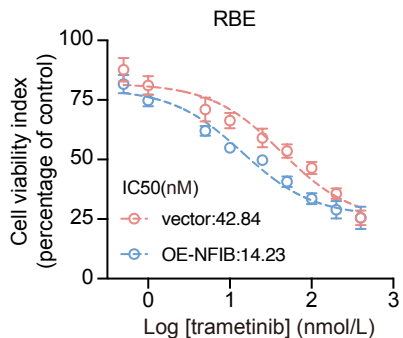

Supplement: Supplementary file 17 — Additional file 17. [file 12943_2021_1482_MOESM17_ESM.pdf]

**Figure S9**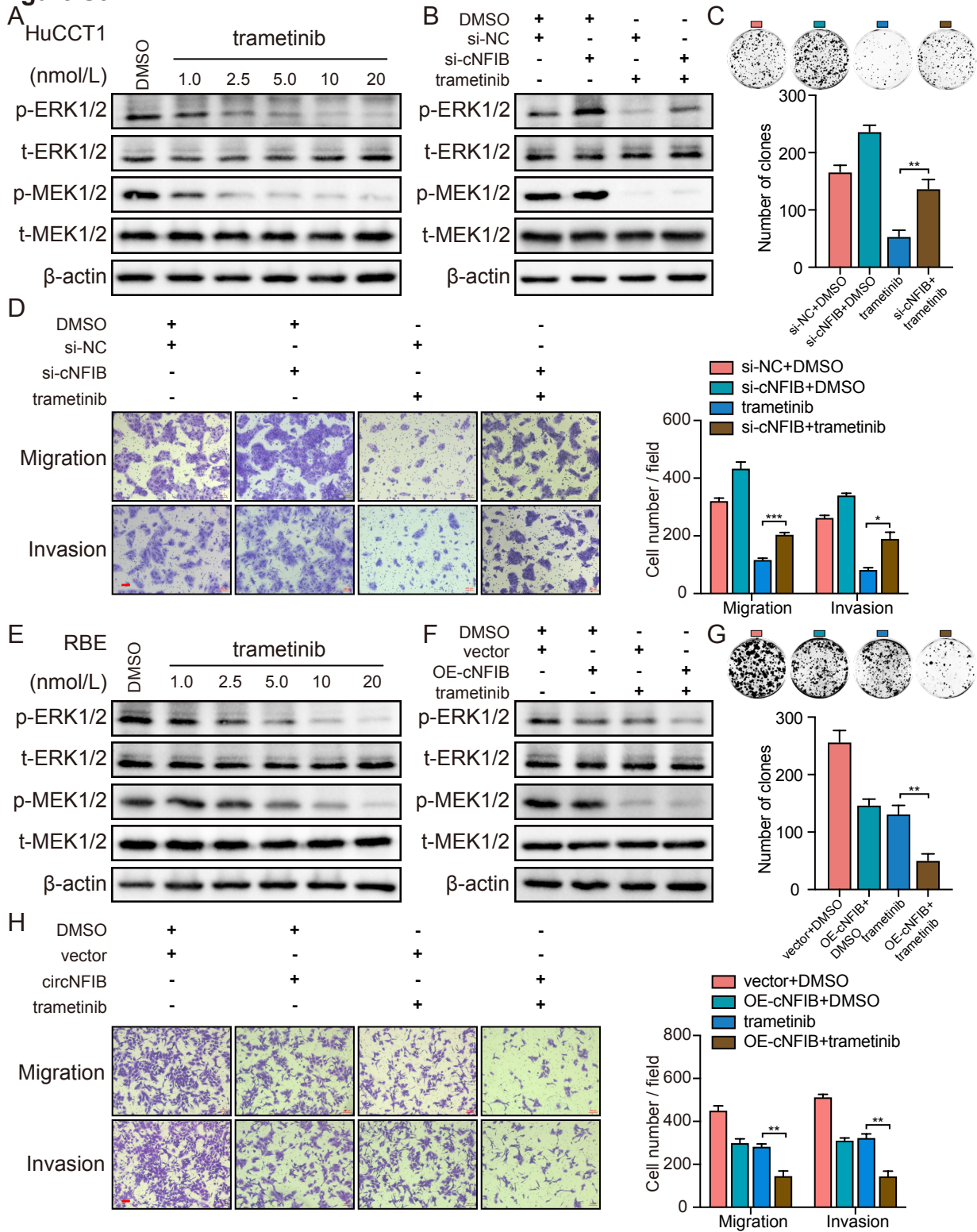

Supplement: Supplementary file 18 — Additional file 18. [file 12943_2021_1482_MOESM18_ESM.pdf]

# Figure S10

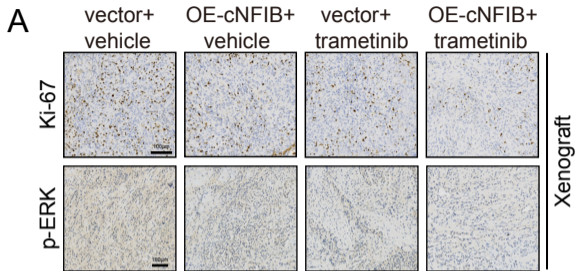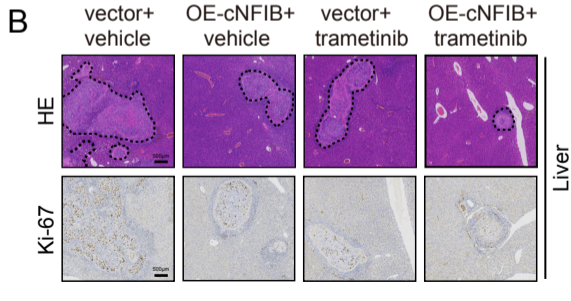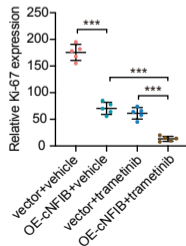

Supplement: Supplementary file 19 — Additional file 19. [file 12943_2021_1482_MOESM19_ESM.pdf]
