# Supplementary material for: CircNFIB inhibits tumor growth and metastasis through suppressing MEK1/ERK signaling in intrahepatic cholangiocarcinoma
Source: Mol Cancer. 2022 Jan 17;21:18. doi: 10.1186/s12943-021-01482-9 (PMC8762882; doi:10.1186/s12943-021-01482-9)
Supplement: Supplementary file 15 — Additional file 15. [file 12943_2021_1482_MOESM15_ESM.pdf]

Figure S6

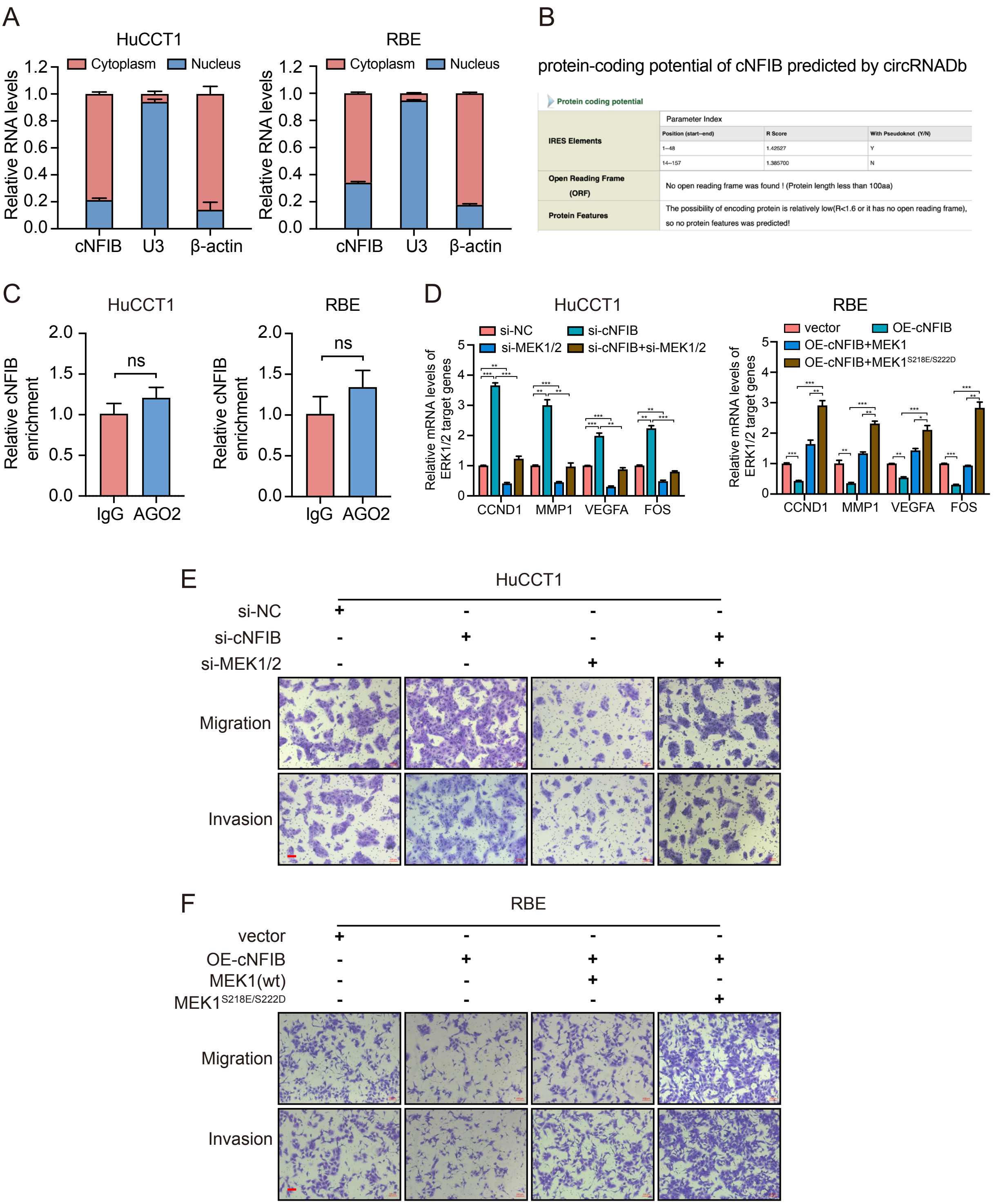
A
HuCCT1
Cytoplasm
Nucleus
Relative RNA levels
cNFIB
U3
β-actin
RBE
B
protein-coding potential of cNFIB predicted by circRNADb
Protein coding potential
Parameter Index
IRES Elements
Position (start--end)
R Score
With Pseudoknot (Y/N)
1--48
1.42527
Y
14--157
1.385700
N
Open Reading Frame (ORF)
No open reading frame was found ! (Protein length less than 100aa)
Protein Features
The possibility of encoding protein is relatively low(R<1.6 or it has no open reading frame), so no protein features was predicted!
C
HuCCT1
RBE
Relative cNFIB enrichment
ns
IgG
AGO2
D
HuCCT1
RBE
si-NC
si-cNFIB
si-MEK1/2
si-cNFIB+si-MEK1/2
vector
OE-cNFIB
OE-cNFIB+MEK1
OE-cNFIB+MEK1S218E/S222D
Relative mRNA levels of ERK1/2 target genes
CCND1
MMP1
VEGFA
FOS
E
HuCCT1
si-NC
si-cNFIB
si-MEK1/2
Migration
Invasion
F
RBE
vector
OE-cNFIB
MEK1(wt)
MEK1S218E/S222D
Migration
Invasion
