# Supplementary material for: CircNFIB inhibits tumor growth and metastasis through suppressing MEK1/ERK signaling in intrahepatic cholangiocarcinoma
Source: Mol Cancer. 2022 Jan 17;21:18. doi: 10.1186/s12943-021-01482-9 (PMC8762882; doi:10.1186/s12943-021-01482-9)
Supplement: Supplementary file 20 — Additional file 20. [file 12943_2021_1482_MOESM20_ESM.docx]

**Supplementary Materials and Methods**

**RNA sequencing**

Before circRNA sequencing, the ribosomal RNA and the linear RNA were removed from total RNA extracted from 30 ICC tissues with ribo-zero-magnetic-kit (Epicentre) and RNase R (Epicentre), respectively. Next, a total of 3 micrograms RNA per sample was used to generate sequencing libraries by using NEBNext® UltraTM RNA Library Prep Kit for Illumina® (NEB, USA) [1]. After that, the samples were sequenced on an Illumina Hiseq platform and 125 bp/150 bp paired-end reads were generated. This program was performed at Novogene Co., Ltd (Beijing, China).

The mRNA sequencing program was performed at LC-Bio Technology co.,ltd (Hangzhou, China). Briefly, HuCCT1 cells were transfected with cNFIB siRNA or the control. Then the total RNAs were isolated from these cells using TRIzol agent (Invitrogen, CA, USA). After establishing sequencing libraries, samples were subjected to perform the 2×150bp paired-end sequencing (PE150) on an Illumina Novaseq™ 6000.

**RNase R treatment**

In brief, 3 micrograms of total RNA were incubated at 37 °C for 30min with 10U RNase R (Epicentre Technology, USA), followed by incubating at 70 °C for 10min to remove the RNase R, and subsequently examined using qRT-PCR.

**Actinomycin D assay**

HuCCT1 and RBE cells were treated with Actinomycin D (2ug/mL) for 0h, 4h, 8h, 12h, 24h, respectively. The expression levels of cNFIB and NFIB mRNA were detected by qRT-PCR.

**Quantitative reverse transcription PCR (qRT-PCR)**

Total RNAs were isolated using Cell Total RNA Isolation Kit (Foregene Biotech, Chengdu, China). 1 microgram of total RNA was used for reverse transcription using HiScript II Q RT SuperMixfor qPCR (+gDNA wiper) (Vazyme Biotech, Nanjing, China). Subsequently, real-time PCR was performed using ChamQ™ SYBR@ qPCR Master Mix (Vazyme Biotech, Nanjing, China). The relative expression of RNA was normalized by β-actin or U6 and quantitated by the 2^−ΔΔ^Ct method. Primers used in this study were shown in Table S6.

**Small interfering RNA transfection, plasmid transfection and lentiviral infection**

Small interfering RNA (siRNA) targeting cNFIB was obtained from RiboBio Co., Ltd. (Guangzhou, China). Genmute^TM^ Reagent (SignaGen Laboratories, Maryland, USA) was used to transfected siRNA according to the manufacturer’s instructions. Plasmid transfection was conducted with GenJet^TM^ plus Reagent (SignaGen Laboratories, Maryland, USA). Cells were harvested at 48h post transfection. The following lentiviral vectors were synthesized by Hanbio (Shanghai, China): cNFIB overexpression (OE-cNFIB) and its control vector (vector); cNFIB knockdown (sh-NFIB) and its control vector (sh-NC). Stable infection was conducted according to standard procedures. The target sequences of siRNAs were listed in Table S7.

**Cell Counting Kit-8 (CCK-8) assay, cell cycle analysis, colony formation assays and EdU (5-Ethynyl-2’-deoxyuridine) labelling assay**

Cell viability was examined by CCK-8 assay (Beyotime Biotechnology, Shanghai, China). In details, 1.5 × 10^3^ cells suspended in 100μl of complete culture media were seeded in 96-well plates and 10μL of CCK-8 solution was added into each well at indicated time point and incubated at 37°C for 2 h, followed by detecting the absorbance of each well using the Eon^TM^ Microplate Reader (BioTek, VT, USA) at wavelength of 450 nm.

Cell cycle was detected by using Cell Cycle Kit (4ABiotech, Beijing, China) according to the manufacturer’s instructions. Cells labeled with PI were subjected to detection using CytoFLEX Research Flow Cytometer (Beckman Coulter, CA, USA), and analyzed by Modfit LT software.

For colony formation assays, cells were plated in 12-well plate at a density of 1.0× 10^3^ per wells. After incubating for 2 weeks, cells were fixed by 4% paraformaldehyde and stained with 0.1% crystal violet. Clone number was calculated by Image J software (National Institutes of Health, Bethesda, MD, USA).

EdU labelling assay was another method to assess cell proliferation. 2.0× 10^4^ cells were planted into 24-well plates in triplicate. EdU (RiboBio Biotechnology, Guangzhou, China) was added into each well and incubated for 2 h, followed by fixing in paraformaldehyde and quenching with glycine solution. After incubation with Apollo dye solution for 30 min, cells were photographed the OBSERVER D1/AX10 cam HRC microscope (Zeiss, Oberkochen, Germany).

**Wound-healing assay and transwell assay**

For wound-healing assay, cells were seeded into 6-well plates with proper density. A homogenous wound was scratch with a 200μL pipette tip when cells reached to 90% cell confluence. After 24h or 48h, images were captured using the OBSERVER D1/AX10 cam HRC microscope (Zeiss, Oberkochen, Germany) and calculated by Image J software.

The transwell chamber (8.0μm pore size, Corning Costar, Kennebunk, USA) coated with (invasion) or without (migration) matrigel was used. For migration assay, 2 × 10^4^ cells suspended in 500μl of serum-free 1640 were planted to the upper chamber in the insert of a 24-well plate. For Matrigel invasion assay, 4 × 10^4^ cells suspended in 500μl of serum-free 1640 were planted to the upper chamber in the insert of a 24-well plate. 1640 medium with 10% FBS was added into the lower chamber. 24h or 48h later, the cells that migrated to the bottom surface of the chamber were fix with paraformaldehyde, stained with crystal violet and captured by microscope.

**RNA FLUORESCENCE IN SITU HYBRIDIZATION (FISH)**

The subcellular location of cNFIB was detected using the FISH kit (RiboBio). Cy3- conjugated probes against cNFIB, U6 snRNA and 18S rRNA were designed and synthesized by RiboBio. FISH assay was performed according to the manufacturer’s protocol of the FISH kit. The images were captured by the A1RþMP Confocal Laser Microscope System (Nikon).

**Subcellular RNA fractionation**

Cytoplasmic and nuclear RNA fractions were isolated by using with the PARISTM Kit

(Invitrogen, CA, USA) according to manufacturer's protocol, followed by qRT-PCR analysis. U3 and β-actin were used as nuclear and cytoplasmic internal reference, respectively.

**circRNA pull-down**

EZ-Magna ChIRP RNA Interactome Kit (Millipore, Massachusetts, USA) was used to performed circRNA pull-down assays. The biotin-labelled cNFIB probes and control probes were synthesized by RiboBio (RiboBio Biotechnology, Guangzhou, China). Formaldehyde-cross-link cells were lysed in complete lysis buffer, followed by incubation with biotin-labelled probes at 37°C overnight. After that, streptavidin magnetic beads were added into lysis buffer incubating at 37°C for 60min so that the ptotein-circRNA-probe complexes could immobilized on the beads. Beads then were magnetically separated and washed 6 times. 20% of RNA-protein complexes were used to purified RNA by TRIzol reagent, which was then subjected to qRT-PCR to quantify the enrichment of cNFIB. The remaining 80% of RNA-protein complexes were used to extract protein, which was subjected to western blot analysis or MS analysis. The biotinylated probes can be found in Table S8.

**RNA immunoprecipitation assays (RIP)**

The Magna RIP^TM^ RNA-binding Protein Immunoprecipitation Kit (Millipore, Massachusetts, USA) was used to perform RIP assay. Briefly, 5ug of specific antibody or normal IgG (Millipore, Massachusetts, USA) in 500μl of lysis buffer containing protease inhibitor cocktail was immobilized on magnetic beads via incubation at 37°C for 60min. Then, cells lysates prepared in the complete lysis buffer were added to tubes with antibody-coated beads and incubated at 4°C overnight. RNA-protein complexes were washed 6 times. 10% of coprecipitated complexes was subjected to western blot analysis to asses immunoprecipitation efficacy of antibodies. 90% of complexes were used to purify RNA with phenol: chloroform: isoamyl alcohol, followed by qRT-PCR analysis to detect the enrichment of cNFIB.

**Co-immunoprecipitation (Co-IP)**

Co-IP was conducted using Pierce Crosslink Magnetic IP/Co-IP Kit in (Thermo Fisher Scientific, CA, USA) following to the manufacturer’s instruction. In brief, precleared cell lysates were incubated with magnetic beads coated with 5ug of antibodies at 4°C overnight, followed by washing the bead complexes 3 times. Washed complexes were then treated with elution buffer, subsequently boiled in protein loading buffer before western blot analysis.

**Proximity ligation assay (PLA)**

PLA was used to identify specific protein-protein interactions through oligonucleotide-conjugated secondary antibodies. When the distance of two different proteins was less than 40nm, fluorescently labeled oligonucleotides could be initiated the amplification of a Texas red reporter signal under certain conditions. PLA was conducted using Duolink® In Situ Red Starter Kit Mouse/Rabbit according to the manufacturer’s protocol (Sigma-Aldrich, USA). Briefly, cells were fixed with 4% paraformaldehyde and permeabilized with 0.2% Triton X-100, after which incubated with blocking solution for 60 min and primary antibodies at 4°C overnight. Next, cells were incubated with secondary antibodies conjugated with oligonucleotides (anti-rabbit PLUS probe and anti-mouse MINUS probe) at 37 °C for 60 min, followed by incubation with ligation-ligase solution at 37°C for 60 min and amplification-polymerase solution at 37°C for 100 min, respectively. Fluorescent signal was visible under the OBSERVER D1/AX10 cam HRC microscope (Zeiss, Oberkochen, Germany). Every distinct fluorescent dot represents the close proximity of two interacting proteins within the cells.

**Immunohistochemistry (IHC)**

Paraffin-embedded tissue-microarrays were deparaffinized, rehydrated followed by antigen retrieval through heat mediation in citrate buffer. Samples were blocked with 5% goat serum and then incubated with primary antibodies at 4°C overnight. DAB solution was used for chromogenic reaction. Quantitative analyses were performed by multiplying the proportion of positively stained tumor cells by the score for staining intensity (SI: 0=negative staining; 1=weak staining; 2=moderate staining; 3=strong staining). The total score of quantitation ranges from 0 to 300.

**Western blot analysis**

Cells were subjected to protein extraction using RIPA Lysis Buffer (Beyotime Biotechnology, Shanghai, China) containing protease inhibitor cocktail (Thermo Fisher Scientific, USA), and then quantification by BCA Protein Assay Kit (Beyotime Biotechnology, Shanghai, China). We performed western blot assay according to previous reports [2]. Antibodies and reagents used in this study could be found in Table S9.

**Animal studies**

Male 5- to 6-week-old BALB/c nude mice were purchased from Vital River Co., Ltd. (Beijing, China). For subcutaneous xenograft models, 5 × 10^6^ stably transfected ICC cells suspended in 100 μL PBS were subcutaneously injected into the right flank of nude mice. Tumor length and width were recorded weekly and tumor volume was calculated as follows: volume (mm^3^) = length x (width)^2^ × 0.52. Five weeks later, mice were euthanized and their tumors were isolated and weighed. For liver orthotopic-implantation models, 2 × 10^6^ ICC cells were implanted into the left lobe of liver after anesthesia. 6 weeks later, tumor formation and metastasis were visualized by IVIS@ Lumina II system (Caliper Life Sciences, Hopkinton, MA, USA) after intraperitoneal injection of D-luciferin (150 mg/kg). 2 × 10^6^ cells were injected into the tail vein to establish the lung metastasis models. 8 weeks later, tumor metastasis in the lung were scanned by IVIS system following D-luciferin injection. Mice were sacrificed after IVIS measurement, tumor tissues were excised for further detection.

To examine the effects of trametinib on tumor growth and metastasis, we first established the 3 models mentioned above by using stable transfection RBE cells. When subcutaneous tumor reached a volume of 100-130 mm^3^, mice were randomized to receive vehicle or trametinib treatment. Mice were dosed orally with trametinib once daily at 1 mg/kg for 15 consecutive days and tumor volume were monitored per 3 days. After establishing liver orthotopic-implantation models and lung metastasis models, mice were administrated trametinib from the 5^th^ week and 7^th^ week, respectively. 15 days later, tumor formation and metastasis were detected by IVIS.

**References**

1. Zhang Y, Zhang XO, Chen T, Xiang JF, Yin QF, Xing YH*, et al.* Circular intronic long noncoding RNAs. Mol Cell **2013**;51:792-806

2. Du J, Zhu Z, Xu L, Chen X, Li X, Lan T*, et al.* ARHGEF11 promotes proliferation and epithelial-mesenchymal transition of hepatocellular carcinoma through activation of β-catenin pathway. Aging (Albany NY) **2020**;12:20235-53
